# Immunomodulatory Effects of Subacute Inhalation Exposure to Copper Oxide Nanoparticles in House Dust Mite-Induced Asthma

**DOI:** 10.1021/acsnano.3c01668

**Published:** 2023-07-18

**Authors:** Sudartip Areecheewakul, Andrea Adamcakova-Dodd, Zeb R. Zacharias, Xuefang Jing, David K. Meyerholz, Kevin L. Legge, Jon C. D. Houtman, Patrick T. O’Shaughnessy, Peter S. Thorne, Aliasger K. Salem

**Affiliations:** † Department of Pharmaceutical Sciences and Experimental Therapeutics, University of Iowa, Iowa City, Iowa 52242, United States; ‡ Department of Occupational and Environmental Health, University of Iowa, Iowa City, Iowa 52242, United States; § Interdisciplinary Immunology Graduate Program, Department of Pathology, University of Iowa, Iowa City, Iowa 52242, United States; ∥ Department of Pathology, University of Iowa, Iowa City, Iowa 52242, United States; ⊥ Department of Microbiology and Immunology, Carver College of Medicine, University of Iowa, Iowa City, Iowa 52242, United States; # Human Toxicology Program, University of Iowa, Iowa City, Iowa 52242, United States

**Keywords:** copper oxide, nanoparticles, immunomodulatory effects, inhalation, pulmonary toxicity, house dust mite asthma model, immunotherapy

## Abstract

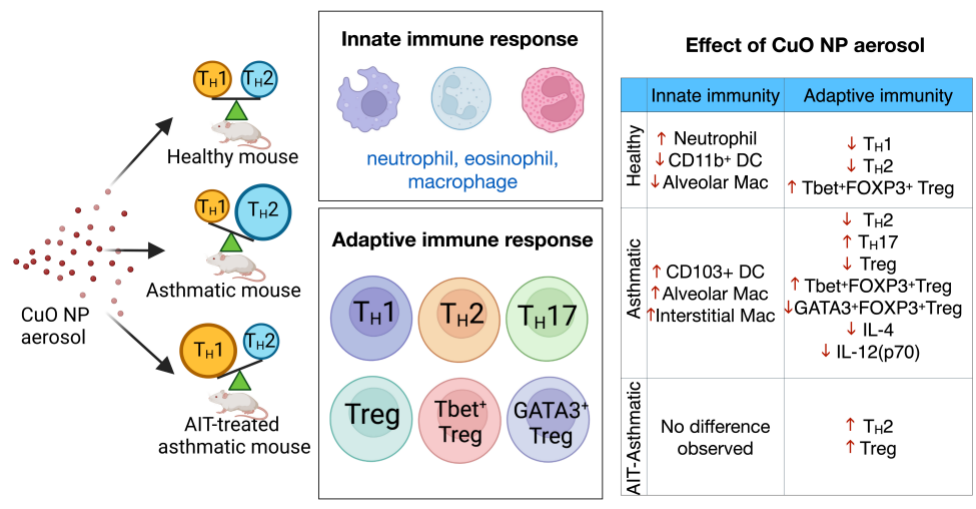

It has been shown that inhalation exposure to copper oxide nanoparticles (CuO NPs) results in pulmonary inflammation. However, immunomodulatory consequences after CuO NP inhalation exposure have been less explored. We tested the effect of CuO NP aerosols on immune responses in healthy, house dust mite (HDM) asthmatic, or allergen immunotherapy (AIT)-treated asthmatic mice (BALB/c, females). The AIT consisted of a vaccine comprising HDM allergens and CpG-loaded nanoparticles (CpG NPs). AIT treatment involved mice being immunized (via subcutaneous (sc) injection; 2 doses) while concomitantly being exposed to CuO NP aerosols (over a 2 week period), starting on the day of the first vaccination. Mice were then sensitized twice by sc injection and subsequently challenged with HDM extract 10 times by intranasal instillation. The asthmatic model followed the same timeline except that no immunizations were administered. All mice were necropsied 24 h after the end of the HDM challenge. CuO NP-exposed healthy mice showed a significant decrease in T_H_1 and T_H_2 cells, and an elevation in T-bet^+^ Treg cells, even 40 days after the last exposure to CuO NPs. Similarly, the CuO NP-exposed HDM asthma model demonstrated decreased T_H_2 responses and increased T-bet^+^ Treg cells. Conversely, CuO NP inhalation exposure to AIT-treated asthmatic mice resulted in an increase in T_H_2 cells. In conclusion, immunomodulatory effects of inhalation exposure to CuO NPs are dependent on immune conditions prior to exposure.

## 1 Introduction

The increased production of metal and metal oxide nanoparticles (NPs), including copper oxide (CuO) NPs creates an increased potential for unintended occupational or consumer inhalation exposure, which may have immunopathogenic consequences, the severity of which may depend on the immune status of the individual exposed. NPs can have a wide range of immunomodulatory effects including promoting polarization of T_H_1 or T_H_2 type responses.^1,2^ It has been shown that various types of NPs can affect dendritic cell (DC) functions, such as cell maturation and homing capability. They may also compromise antigen processing and presentation, and can affect DC-induced T cell differentiation.^3^*In vitro* studies with gold (Au) NPs found size-dependent effects on DCs, where 10 nm Au NPs inhibited LPS-induced production of IL-12p70 and potentiated their T_H_2 polarization capacity, while 50 nm Au NPs promoted T_H_17 polarization.^4^ ZnO NPs administered by intraperitoneal injection to BALB/c mice provided an adjuvant effect to a T_H_2 response^5^ to ovalbumin (OVA) through the activation of Toll-like receptors-2, -4 and -6.^6^

Previous studies by us and others have demonstrated pulmonary toxicity induced by inhalation exposure to CuO NPs in healthy mice.^7−9^ Immediately after acute and subacute inhalation exposure to CuO NP aerosols, strong airway inflammation and cytotoxicity occurred as indicated by increased cell infiltration (primarily neutrophils) and increased levels of lactate dehydrogenase (LDH) in the bronchoalveolar lavage (BAL) fluid. Continuous three-month exposure to inhaled CuO NPs in healthy mice has shown that CuO NPs mainly influenced innate immune cells with a minimal effect on T and B lymphocytes in the spleen.^10^ However, CuO NP exposure can affect immune responses differently when compared to those under pathological versus healthy conditions. Mice first exposed to Cu NPs via inhalation or intratracheal instillation and then inoculated with *Klebsiella pneumoniae* exhibited impaired host defenses against bacterial infections of the lungs due to decreased bacterial clearance that correlated with increased Cu NP concentration exposure.^11^

Allergic asthma, a chronic lung disease characterized by reversible airway obstruction and inflammation, is primarily caused by type 2 immune responses. Allergic airway diseases, such as asthma, have been studied in mouse models to assess the impact of inhalation exposure to CuO NPs, mainly by assessing whether they aggravate or attenuate airway inflammation and airway hyper-responsiveness (AHR). Mice sensitized with OVA by intraperitoneal injection and then given CuO NPs by intranasal instillation during the OVA challenge showed that CuO NPs aggravated the development of asthma by enhancing AHR, inflammatory cell counts in BAL fluid, mucus secretion, serum immunoglobulin E (IgE) levels, and allergic inflammatory markers (including IL-5, IL-13, and reactive oxygen species (ROS) production) in BAL fluid.^12^ Kooter et al. used an air–liquid interface system to investigate responses to CuO NP aerosols in three-dimensional human bronchial epithelia isolated from healthy and asthmatic subjects. The cells derived from asthmatic subjects had increased sensitivity to CuO NP aerosols as indicated by increased LDH-based cytotoxicity and increased production of proinflammatory cytokines, such as IL-6, IL-8 and MCP-1, which might be the cause of hyper-reactive airways and insufficient mucociliary clearance in asthmatic patients.^13^

To prevent allergic airway diseases, allergen immunotherapy (AIT) has been developed to induce immunological tolerance and is recommended as a means of managing allergic diseases.^14^ AIT affects both T cell and B cell responses to allergens (e.g., minimizes T_H_2-skewed immune responses in the lung; decreases allergen-specific IgE levels in serum).^14^ The impact of CuO NP inhalation exposure on asthmatic or AIT-treated asthmatic mice could differ based on the model used. It may aggravate AHR as it was found when asthmatic mice were exposed to CuO NPs during OVA challenge by intranasal instillation.^12^ However, to the best of our knowledge, there has been no study performed to date on the effects of CuO NP inhalation exposure in a developing asthmatic mouse model (CuO NPs exposure prior to allergen sensitization) and an AIT-treated asthmatic model.

**Figure 1:**
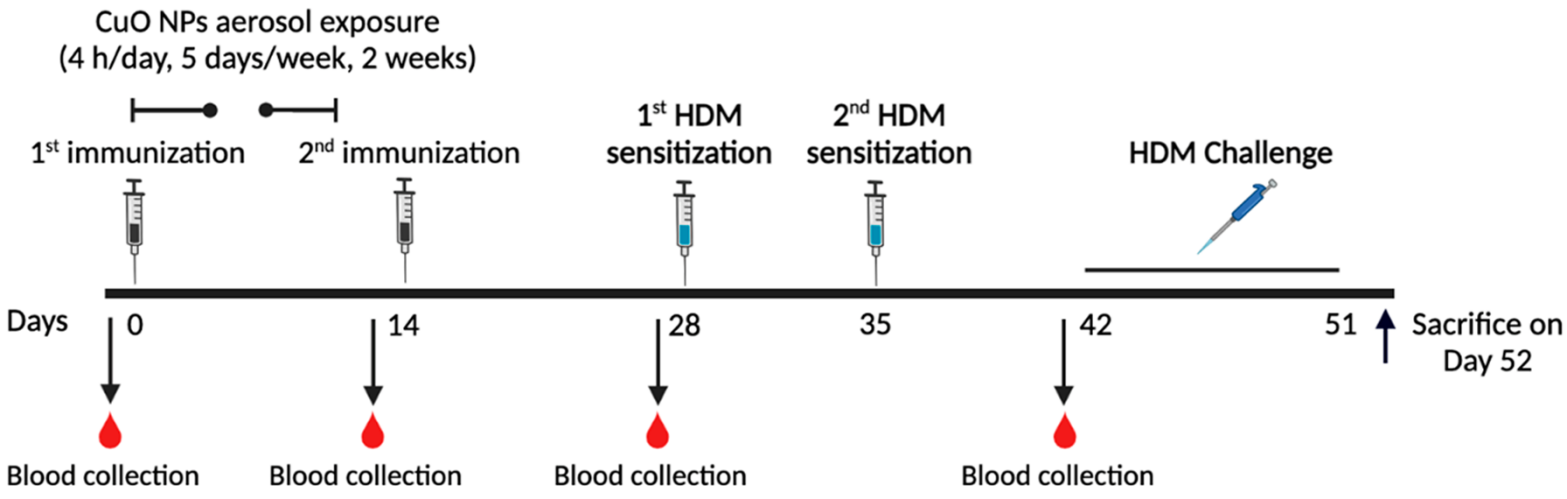
Schematic showing an experimental timeline for immunization and CuO NP inhalation exposure in an asthmatic mouse model. Mice were immunized using AIT comprising 100 μg of Der p1/Der p2 with 50 μg of CpG NPs in 150 μL of saline on days 0 and 14. On day 0, after first immunization, mice were exposed to CuO NP aerosols for 4 h/day, 5 days/week for 2 weeks. Mice were then sensitized with 100 μg of HDM extracts in 100 μL of saline by subcutaneous injection on days 28 and 35, and then challenged with 25 μg of HDM extracts in 50 μL of saline by intranasal instillation on days 42 to 51 consecutively. The asthmatic model followed the same timeline, except that no immunizations were administered. Blood collection by retro-orbital bleeding was performed on days 0, 13, and 28 to measure serum HDM-specific immunoglobulin levels (e.g., IgE, IgG_1_, and IgG_2a_). On day 52, mice were euthanized to performed cellular analysis of BAL fluid to identify immune cells (e.g., eosinophils, neutrophils, macrophages, lymphocytes) as well as measure interstitial CD4^+^ T cell subset levels (e.g., T_H_1, T_H_2, T_H_17, Treg cells), and interstitial immune cell levels (eosinophils, neutrophils, AMs, IMs, CD103^+^ DCs, CD11b^+^ DCs) in the lung homogenates, serum HDM-specific immunoglobulin levels, and AHR.

In this study, we investigated the effect of inhalation exposure to CuO NP aerosols in three distinct immune contexts: naïve (immune homeostasis); asthmatic (dominant T_H_2 responses); and AIT-treated asthmatic (dominant T_H_1 responses) mouse models. We performed cellular analysis of BAL fluid to identify and enumerate immune cells (e.g., eosinophils, neutrophils, macrophages, lymphocytes) and measured levels of interstitial CD4^+^ T cell subsets (e.g., T_H_1, T_H_2, T_H_17, Treg cells) and interstitial innate immune cells (e.g., eosinophils, neutrophils, alveolar macrophages (AMs), interstitial macrophages (IMs), CD103^+^ DCs, CD11b^+^ DCs) in the lung homogenates, as well as measured house dust mite (HDM)-specific immunoglobulin levels (IgE, IgG_1_, and IgG_2a_) in serum, AHR, and lung histopathology. The AIT used in this study consisted of HDM allergens and CpG-loaded nanoparticles (CpG NPs). To model CuO NP-exposed AIT-treated asthmatic mice, mice were immunized with 2 doses of purified *Dermatophagoides pteronyssinus* 1 and 2 antigen (Der p 1 and 2) + CpG NPs by subcutaneous (sc) injection and also exposed to CuO NP aerosols over a 2 week period, starting on the first immunization day. Mice were then sensitized twice by sc injection and subsequently challenged 10 times by intranasal instillation with HDM extracts (see Figure 1 for a schematic describing the timeline). The asthmatic mouse model followed the same timeline, except no immunizations were performed which allowed us to investigate the effect of inhalation exposure to CuO NP aerosols on developing HDM-induced asthma. To characterize immune responses in each model and to demonstrate that the asthmatic and AIT models indeed differed in T_H_2- and T_H_1-dominated responses (respectively), comparisons of measured end points between these two models as well as between CuO NP-exposed and healthy model are also shown. Investigating the effects of CuO NP inhalation exposure under three different conditions (i.e., naïve/healthy, asthmatic, AIT-treated asthmatic mice) provides a deeper understanding of the immunomodulatory effects of CuO NP aerosols on both innate and adaptive immune responses.

## 2 Results

### 2.1 Characterization of CpG NPs and Quantification of CpG Loading

**Figure 2:**
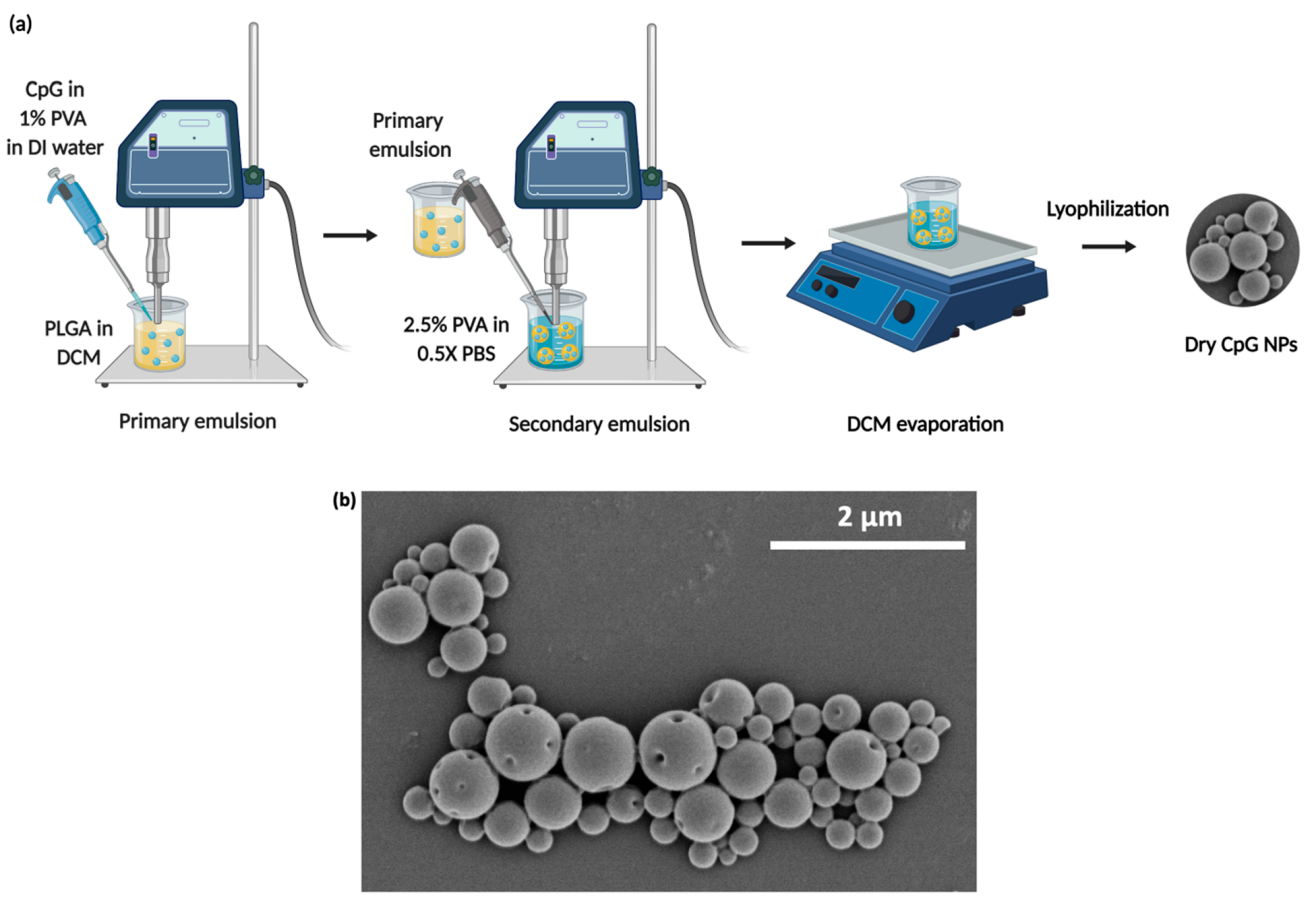
(a) Schematic of CpG NP preparation using a double emulsion solvent evaporation technique. (b) SEM image of CpG NPs.

The hydrodynamic diameter and zeta potential of CpG NPs were 577 ± 6 nm (with a polydispersity index of 0.21) and −29.4 mV, respectively. According to scanning electron microscopy (SEM) images of NPs, the primary particle size was 391 ± 170 nm (*n* = 50), and the NPs were spherical in shape (Figure 2-b). CpG loading was 4.56 μg/mg of particles with 12.5% encapsulation efficiency.

### 2.2 CuO NP Aerosol Characterization

Mice were exposed to CuO NPs by nose-only inhalation for 10 days over a 2 week period (see Materials and Methods). The aerosol size distribution had a geometric mean (GM) mobility diameter of 32.3 nm with a geometric standard deviation (GSD) of 1.7 (Figure S1), demonstrating minimal agglomeration. This result is similar to our previous inhalation study using the same material, which showed a GM (GSD) of 33.3 (1.7) nm.^15^

### 2.3 Establishment of Asthmatic Mouse Model and Allergen Immunotherapy (AIT)-Treated Asthmatic Mouse Model

#### 2.3.1 Asthmatic Mouse Model

**Figure 3:**
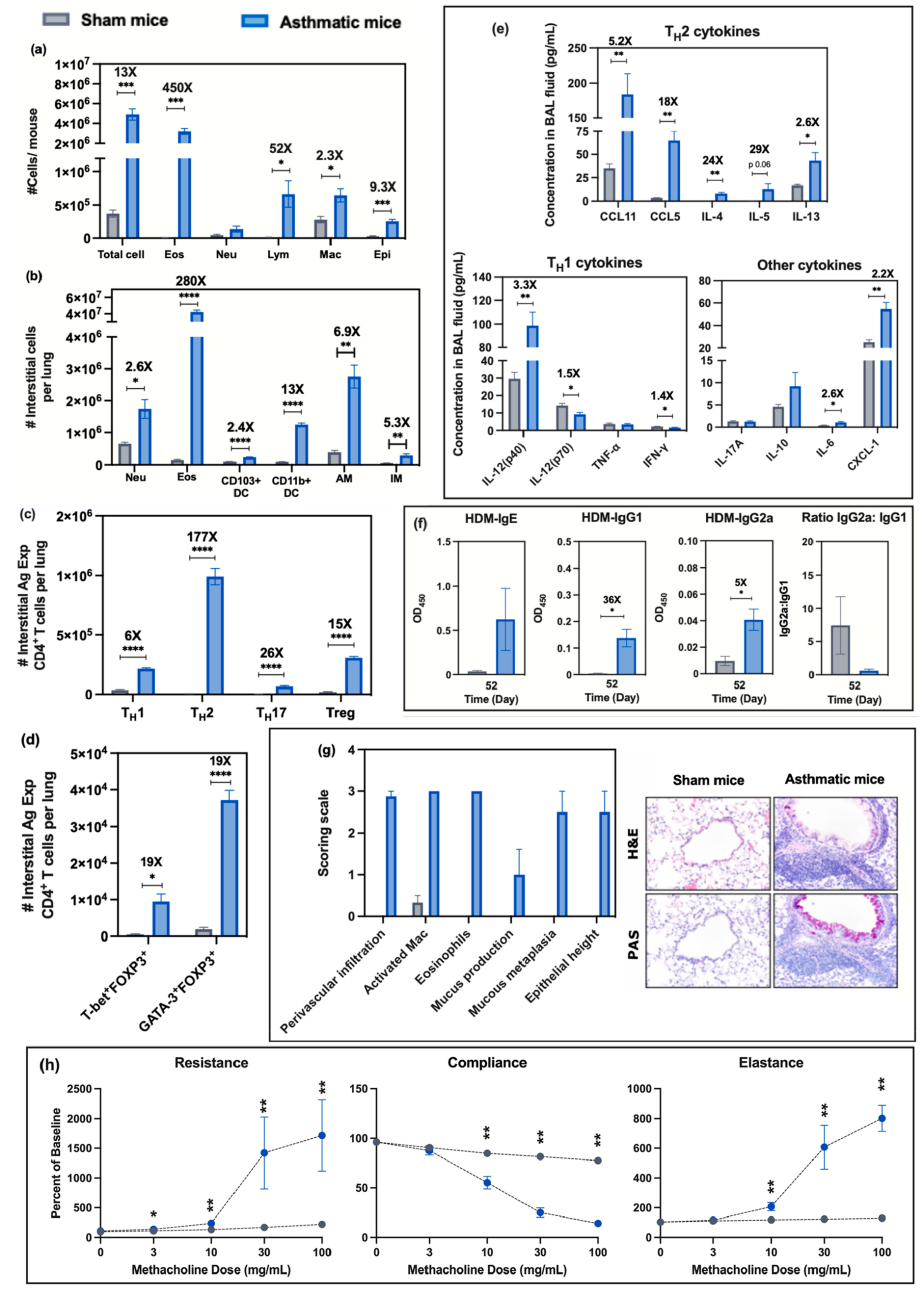
Assessment of asthmatic mouse model compared to sham mice (comparison a): (a) cellular analysis of BAL fluid including total cell number, as well as the number of eosinophils (Eos), neutrophils (Neu), lymphocytes (Lym), macrophages (Mac), and epithelial cells (Epi); (b) numbers of indicated interstitial cells including neutrophils (Neu), eosinophils (Eos), CD103^+^ dendritic cells (DCs), CD11b^+^ DCs, AMs, and IMs in lung tissue homogenates; (c) numbers of interstitial antigen-experienced CD4^+^ T cells including T_H_1, T_H_2, T_H_17, and Treg cells in lung tissue homogenates; (d) number of interstitial antigen-experienced Treg cells expressing either T-bet or GATA3; (e) cytokines/chemokines from BAL fluid measured by Bio-Plex ProTM mouse cytokine 23-plex assay; (f) serum HDM-specific immunoglobulin (IgE, IgG_1_, IgG_2a_) levels and ratio of IgG_2a_:IgG_1_ measured by an indirect ELISA technique on days 13, 27, and 52; (g) representative micrographs of lung sections from each experimental group after H&E staining (upper row) or Periodic Acid-Schiff (PAS, lower row) staining (magnification 20x). Bar graphs show the scoring scale (0, within the scope of normal; 1, rare, but detectable change; 2, mild in distribution/severity; 3, moderate in distribution/severity; 4, severe in distribution/severity) for 6 parameters including increases in the number of activated macrophages, eosinophils, perivascular infiltration (infiltration of lymphocytes around vessels), mucus production, mucous metaplasia, and epithelial height; (h) pulmonary mechanics measurements of mice to assess AHR. Resistance, compliance, and elastance were measured after the inhalation challenge to increase concentrations of methacholine (0, 3, 10, 30, and 100 mg/mL). Statistical analysis was performed using the unequal variance unpaired *t* test (Welch *t* test). Data are shown as mean ± SE (*n* = 6). ****p < 0.0001, ***p < 0.001, **p < 0.01, *p < 0.05.

Cellular analysis of BAL fluid showed a significantly higher level of total cells (*p* < 0.001), eosinophils (*p* < 0.001), lymphocytes (*p* < 0.05), macrophages (*p* < 0.05), and epithelial cells (*p* < 0.001) compared to the sham mice (Figure 3-a). The percentages of eosinophils and lymphocytes in the BAL fluid from the asthmatic mice also showed significant increases (Figure S4-a, *p* < 0.0001 for eosinophils and *p* < 0.01 for lymphocytes), while the percentages of macrophages in asthmatic mice were significantly lower (Figure S4-a, *p* < 0.0001) compared to the sham mice. The significant decrease in the percentage of macrophages in the BAL fluid of asthmatic mice compared to the sham mice corresponded to substantial increases in eosinophils and lymphocytes (Figure S4-a). Lung homogenates from the asthmatic mice had significantly higher total cell numbers (Figure 8, *p* < 0.05) as well as higher numbers of neutrophils (*p* < 0.05), eosinophils (*p* < 0.0001), CD103^+^ DCs (*p* < 0.0001), CD11b^+^ DCs (*p* < 0.0001), AMs (*p* < 0.01), and IMs (*p* < 0.01) compared to the sham mice (Figure 3-b). However, cellular distribution represented by percentages showed significantly higher eosinophils (*p* < 0.0001) with decreases in CD103^+^ DCs (*p* < 0.01), neutrophils (*p* < 0.05), and IMs (*p* < 0.01) (Figure S4-b). The lungs from asthmatic mice had significantly higher numbers of all interstitial antigen-experienced CD4^+^ T cell subsets, including T_H_1 (*p* < 0.0001), T_H_2 (*p* < 0.0001), T_H_17 (*p* < 0.0001), and Treg cells (*p* < 0.0001) compared to the sham mice (Figure 3-c). The percentages of these interstitial antigen-experienced CD4^+^ T cell subsets that could be identified as specific CD4 T cell subsets also showed significant increases for T_H_2 (*p* < 0.0001), T_H_17 (*p* < 0.001), and Treg cells (*p* < 0.001)) compared to the sham mice. No change was observed in T_H_1s (Figure S4-c). Within the Treg cell (i.e., CD4^+^ Foxp3^+^) compartment, asthmatic mice exhibited significant increases in T-bet^+^ cells (*p* < 0.05) and GATA-3^+^ cells (*p* < 0.0001) (Figure 3-d) compared to the sham mice, which concurred with the T-bet^+^ T cells (T_H_1 cells) and GATA-3^+^ T cells (T_H_2 cells) data (Figure 3-c).

The BAL fluid of asthmatic mice exhibited significantly higher levels of all T_H_2 cytokines (CCL11 (*p* < 0.01), CCL5 (*P* < 0.01), IL-4 (*p* < 0.01), and IL-13 (*p* < 0.05)), with the exception of IL-5 (*p* = 0.06), while the T_H_1 cytokines, IL-12(p70) and IFN-γ, were at significantly lower levels (*p* < 0.05). In contrast, the IL-12(p40) subunit, which can function as an inhibitor of IL-12(p70) signaling when it is expressed in a dimer form, was significantly higher compared to BAL fluid from the sham mice (*p* < 0.01) (Figure 3-e). IL-6 and CXCL-1 levels in the BAL fluid from asthmatic mice were significantly higher compared to the sham mice (*p* < 0.05 and *p* < 0.01, Figure 3-e).

The sera from asthmatic mice (collected on day 52) showed a trend toward higher levels of HDM-specific IgE than the sham mice (*p* = 0.16), while showing significantly higher levels of HDM-specific IgG_1_ (*p* < 0.05) and IgG_2a_ (*p* < 0.05) (Figure 3-f). However, the ratio of IgG_2a_:IgG_1_ for the HDM-specific IgG from the sera of asthmatic mice trended lower than that for the sham mice (Figure 3-f). Lung histopathology of asthmatic mice showed increases in all 6 parameters measured, including increases in numbers of eosinophils and activated macrophages, as well as increased perivascular infiltration, mucus production, mucous metaplasia, and epithelial height (which are indicators of asthmatic conditions), compared to sham mice (Figure 3-g). The measurement of pulmonary mechanics in asthmatic mice demonstrated significant increases in resistance at 3 (*p* < 0.05) and 10 (*p* < 0.05) mg/mL methacholine and significant increases in elastance at 10 (*p* < 0.05), 30 (*p* < 0.05), and 100 (*p* < 0.001) mg/mL methacholine, whereas significant decreases in compliance at 10 (*p* < 0.01), 30 (*p* < 0.0001), and 100 (*p* < 0.0001) mg/mL methacholine were observed when compared to the sham group (Figure 3-h).

#### 2.3.2 AIT-Treated Asthmatic Mouse Model

**Figure 4:**
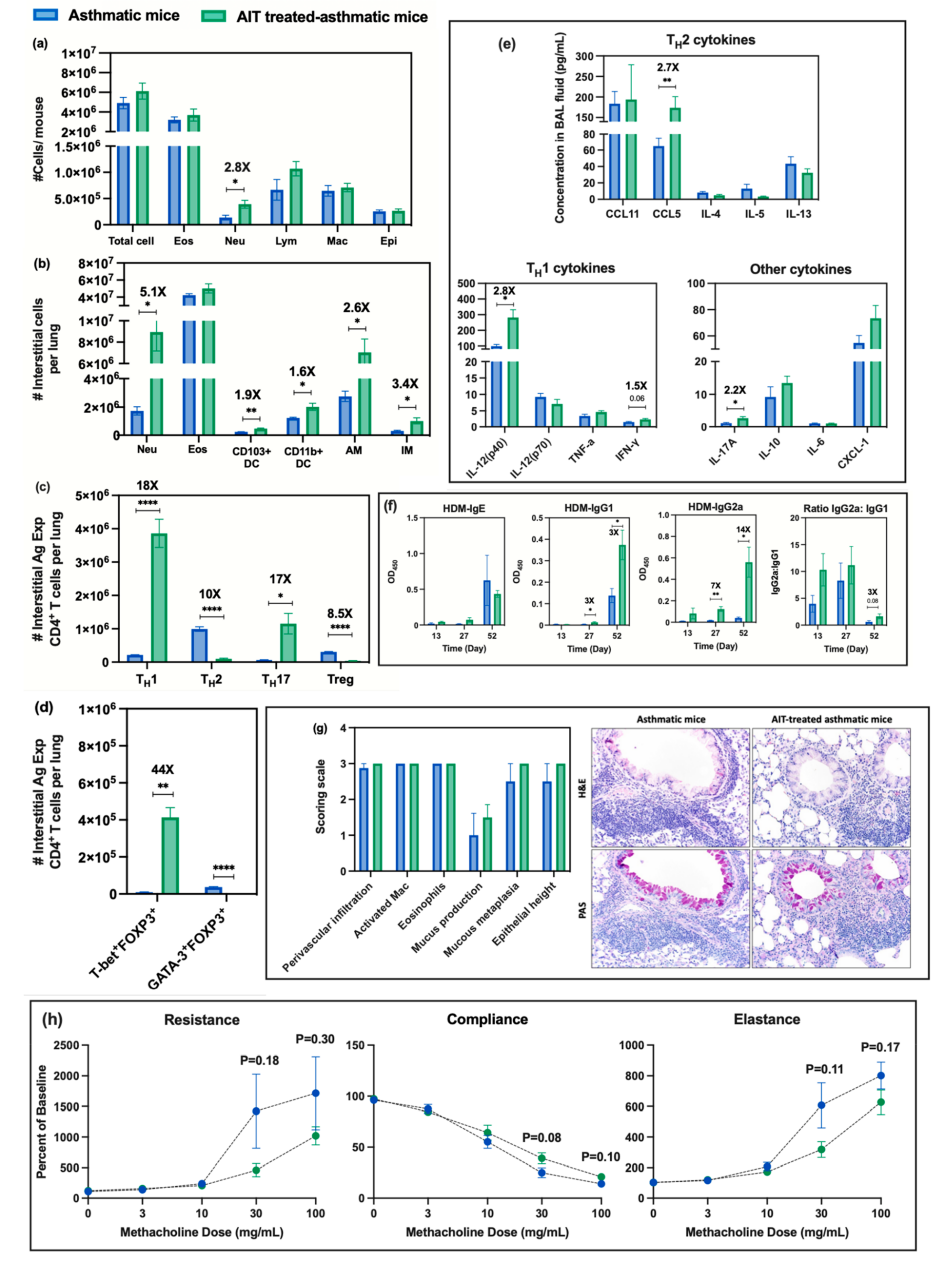
Assessment of AIT-treated asthmatic mice model compared to asthmatic mice (comparison b): (a) cellular analysis of BAL fluid including total cell number, as well as the number of eosinophils (Eos), neutrophils (Neu), lymphocytes (Lym), macrophages (Mac), and epithelial cells (Epi); (b) numbers of indicated interstitial cells including neutrophils (Neu), eosinophils (Eos), CD103^+^ dendritic cells (DCs), CD11b^+^ DCs, alveolar macrophages (AM), and interstitial macrophages (IM) in lung tissue homogenates; (c) numbers of interstitial antigen-experienced CD4^+^ T cells including T_H_1, T_H_2, T_H_17, and Treg cells in lung tissue homogenates; (d) number of interstitial antigen-experienced Treg cells expressing either T-bet or GATA3; (e) cytokines/chemokines from BAL fluid measured by Bio-Plex ProTM mouse cytokine 23-plex assay; (f) serum HDM-specific immunoglobulin (IgE, IgG_1_, IgG_2a_) levels and ratio of IgG_2a_:IgG_1_ measured using an indirect ELISA technique at days 13, 27, and 52; (g) representative micrographs of lung sections from each experimental group after H&E staining (upper row) or Periodic Acid-Schiff (PAS, lower row) staining (magnification 20x). Bar graphs show the scoring scale (0, within the scope of normal; 1, rare, but detectable change; 2, mild in distribution/severity; 3, moderate in distribution/severity; 4, severe in distribution/severity) for 6 parameters including increases in the number of activated macrophages, eosinophils, perivascular infiltration (infiltration of lymphocytes around vessels), mucus production, mucous metaplasia, and epithelial height; (h) pulmonary mechanics measurements of mice to assess AHR. Resistance, compliance, and elastance were measured after the inhalation challenge to increase concentration of methacholine (0, 3, 10, 30, and 100 mg/mL). Statistical analysis was performed using the unequal variance unpaired *t* test (Welch *t* test). Data are shown as mean ± SE (*n* = 6). ****p < 0.0001, ***p < 0.001, **p < 0.01, *p < 0.05.

The number and percentage of neutrophils in the BAL fluid from AIT-treated asthmatic mice were significantly higher compared to the asthmatic mice model (Figure 4-a (*p* < 0.05), Figure S5-a (*p* < 0.05)), while there were no significant differences in the cell number for other cell types. Lung homogenates from the AIT-treated asthmatic mice did not demonstrate statistically significant differences in total cell numbers compared with the asthmatic mice (Figure 8). However, there were significant increases in the numbers of each cell type with the exception of eosinophils (Figure 4-b, *p* < 0.05 for neutrophils, CD11b^+^ DC, alveolar macrophages (AM), and interstitial macrophages (IM); and *p* < 0.01 for CD103^+^ DC). In addition, the percentage of eosinophils in the lung homogenates from the AIT-treated asthmatic mice was significantly lower compared to the asthmatic mice (Figure S5-b, *p* < 0.05). The lung homogenates from AIT-treated asthmatic mice had a significantly higher number of interstitial antigen-experienced CD4^+^ T cells for T_H_1 (*p* < 0.0001) and T_H_17 (*p* < 0.05), while there were significant decreases in T_H_2 (*p* < 0.0001) and Treg cells (*p* < 0.0001) compared to the asthmatic group (Figure 4-c). Similarly, the percentages of antigen-experienced CD4^+^ T cells subsets that could be identified as specific CD4 T cell subsets in AIT-treated asthmatic mice showed highly significant increases in T_H_1 (*p* < 0.0001) and T_H_17 (*p* < 0.01) cells, while decreases were noted for antigen-experienced CD4^+^ T_H_2 (*p* < 0.0001) and Treg cells (*p* < 0.0001) compared to the asthmatic mice (Figure S5-c). Treg cells expressing T-bet^+^ were significantly increased (*p* < 0.01), while there was a significant decrease in Treg cells expressing GATA-3^+^ (*p* < 0.0001) in AIT-treated asthmatic mice compared to asthmatic mice (Figure 4-d). These changes align with the aforementioned data for overall T_H_1 cells and T_H_2 cell responses (Figure 4-c).

BAL fluid from AIT-treated asthmatic mice had significantly higher levels of CCL5 (*p* < 0.01), IL-12(p40) (*p* < 0.05), and IL-17A (*p* < 0.05) than in BAL fluid from asthmatic mice (Figure 4-e). The sera from AIT-treated asthmatic mice showed no significant change in the levels of HDM-specific IgE at all time points when compared to those of asthmatic mice (Figure 4-f). The ratio of IgG_2a_:IgG_1_ for HDM-specific IgG from the sera of AIT-treated asthmatic mice exhibited a trend toward being higher than that for asthmatic mice on day 52 (Figure 4-f, *p* = 0.08). Lung histopathology of AIT-treated asthmatic mice showed no differences in any parameters compared to those of asthmatic mice (Figure 4-g). The AIT-treated asthmatic mice and asthmatic mice showed no significant differences in pulmonary mechanics outcomes; however, the AIT-treated asthmatic group exhibited trends of slightly increased lung compliance (*p* = 0.08 and *p* = 0.10 at 30 and 100 mg/mL methacholine, respectively) (Figure 4-h).

### 2.4 Immunomodulatory Effects of CuO NP Inhalation Exposure to Different Mouse Models

#### 2.4.1 CuO NP Exposure to Healthy Mice

**Figure 5:**
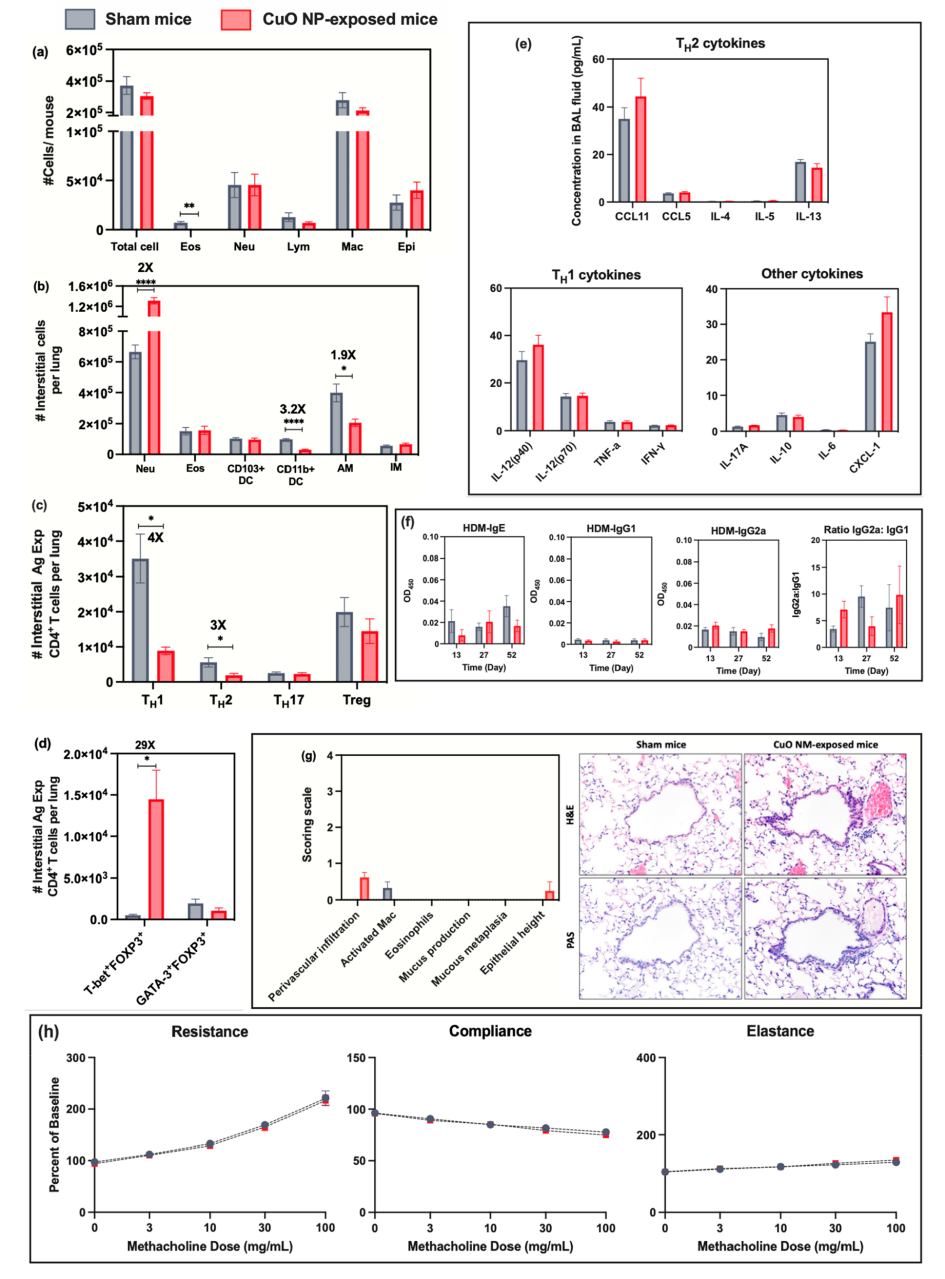
Assessment of immunomodulatory effects of CuO NP inhalation exposure to naive mice 40 days after inhalation exposure (comparison c): (a) cellular analysis of BAL fluid including total cell number, as well as the number of eosinophils (Eos), neutrophils (Neu), lymphocytes (Lym), macrophages (Mac), and epithelial cells (Epi); (b) numbers of indicated interstitial cells including neutrophils (Neu), eosinophils (Eos), CD103^+^ dendritic cells (DCs), CD11b^+^ DCs, alveolar macrophages (AM), and interstitial macrophages (IM) in lung tissue homogenates; (c) numbers of interstitial antigen-experienced CD4^+^ T cells including T_H_1, T_H_2, T_H_17, and Treg cells in lung tissue homogenates; (d) number of interstitial antigen-experienced Treg cells expressing either T-bet or GATA3; (e) cytokines/chemokines from BAL fluid measured by Bio-Plex ProTM mouse cytokine 23-plex assay; (f) serum HDM-specific immunoglobulin (IgE, IgG_1_, IgG_2a_) levels and ratio of IgG_2a_:IgG_1_ measured using an indirect ELISA technique at days 13, 27, and 52; (g) representative micrographs of lung sections from each experimental group after H&E staining (upper row) or Periodic Acid-Schiff (PAS, lower row) staining (magnification 20x). Bar graphs show the scoring scale (0, within the scope of normal; 1, rare, but detectable change; 2, mild in distribution/severity; 3, moderate in distribution/severity; 4, severe in distribution/severity) for 6 parameters including increases in the number of activated macrophages, eosinophils, perivascular infiltration (infiltration of lymphocytes around vessels), mucus production, mucous metaplasia, and epithelial height; (h) pulmonary mechanics measurements of mice to assess AHR. Resistance, compliance, and elastance were measured after the inhalation challenge to increase concentrations of methacholine (0, 3, 10, 30, and 100 mg/mL). Statistical analysis was performed using the unequal variance unpaired *t* test (Welch *t* test). Data are shown as mean ± SE (*n* = 6). ****p < 0.0001, ***p < 0.001, **p < 0.01, *p < 0.05.

At 40 days postexposure, CuO NP-exposed mice showed no significant residual differences in any cell types in BAL fluid compared to the sham mice, except for the number and percentage of eosinophils which were marginally lower compared to BAL fluid from the sham mice (Figure 5-a (*p* < 0.01), Figure S6-a (*p* < 0.001)). Lung homogenates from the CuO NP-exposed healthy mice did not possess significantly higher total cell numbers than did the sham mice (Figure 8). However, CuO NP exposure to healthy mice elicited a significantly higher number of neutrophils (*p* < 0.0001) and resulted in significantly lower numbers of CD11b^+^ DCs (*p* < 0.0001) and AMs (*p* < 0.05) compared to the sham mice (Figure 5-b). The CuO NP-exposed mice showed significantly lower numbers and percentages of interstitial T_H_1 cells (*p* < 0.05) and T_H_2 cells (*p* < 0.05) in lung homogenates compared to the sham mice (Figure 5-c, Figure S6-c). Interestingly, the numbers of antigen-experienced CD4^+^ Treg cells coexpressing T-bet and Foxp3 significantly increased in CuO NP-exposed mice compared to the sham mice (*p* < 0.05, Figure 5-d). Meanwhile, cytokine/chemokine levels in BAL fluid, serum IgE levels, serum IgG_1_ levels, serum IgG_2a_ levels, lung histopathology, and pulmonary mechanics of CuO NP-exposed mice showed no significant residual differences when compared to those in the sham mice (Figure 5-e–h).

#### 2.4.2 CuO NP Exposure to Asthmatic Mice

**Figure 6:**
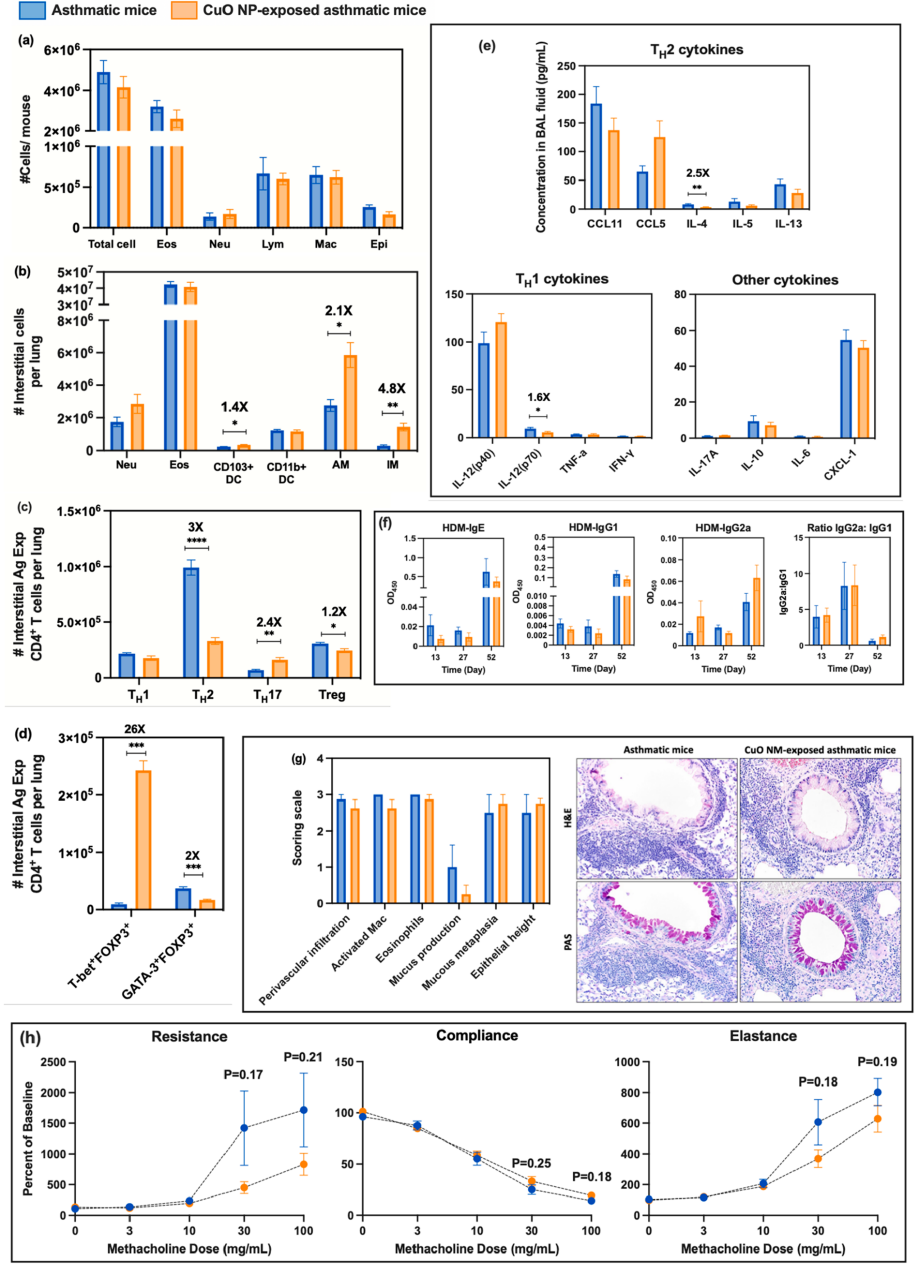
Assessment of immunomodulatory effects of CuO NP inhalation exposure to asthmatic mice 40 day post NP exposure (comparison d): (a) cellular analysis of BAL fluid including total cell number, as well as the number of eosinophils (Eos), neutrophils (Neu), lymphocytes (Lym), macrophages (Mac), and epithelial cells (Epi); (b) numbers of indicated interstitial cells including neutrophils (Neu), eosinophils (Eos), CD103^+^ dendritic cells (DCs), CD11b^+^ DCs, alveolar macrophages (AM), and interstitial macrophages (IM) in lung tissue homogenates; (c) numbers of interstitial antigen-experienced CD4^+^ T cells including T_H_1, T_H_2, T_H_17, and Treg cells in lung tissue homogenates; (d) number of interstitial antigen-experienced Treg cells expressing either T-bet or GATA3; (e) cytokines/chemokines from BAL fluid measured by Bio-Plex ProTM mouse cytokine 23-plex assay; (f) serum HDM-specific immunoglobulin (IgE, IgG_1_, IgG_2a_) levels and ratio of IgG_2a_:IgG_1_ measured by an indirect ELISA technique measured at days 13, 27, and 52; (g) representative micrographs of lung sections from each experimental group after H&E staining (upper row) or Periodic Acid-Schiff (PAS, lower row) staining (magnification 20x). Bar graphs show the scoring scale (0, within the scope of normal; 1, rare, but detectable change; 2, mild in distribution/severity; 3, moderate in distribution/severity; 4, severe in distribution/severity) for 6 parameters including increases in the number of activated macrophages, eosinophils, perivascular infiltration (infiltration of lymphocytes around vessels), mucus production, mucous metaplasia, and epithelial height; (h) pulmonary mechanics measurements of mice to assess AHR. Resistance, compliance, and elastance were measured after the inhalation challenge to increase concentrations of methacholine (0, 3, 10, 30, and 100 mg/mL). Statistical analysis was performed using the unequal variance unpaired *t* test (Welch *t* test). Data are shown as mean ± SE (*n* = 6). ****p < 0.0001, ***p < 0.001, **p < 0.01, *p < 0.05.

There was no significant difference in the numbers and percentages in any cell types in the BAL fluid from the asthmatic mice compared with the CuO NP-exposed asthmatic mice (Figure 6-a, Figure S7-a). The lung homogenates from CuO NP-exposed asthmatic mice did not exhibit significantly different total cell numbers compared to the lung homogenates from asthmatic mice (Figure 8); however, significantly higher numbers of CD103^+^ DCs (*p* < 0.05), AMs (*p* < 0.05), and IMs (*p* < 0.01) were observed in the lung homogenates of CuO NP-exposed asthmatic mice compared to non-CuO NPs exposed asthmatic mice (Figure 6-b). We found that the lung homogenates from CuO NP-exposed asthmatic mice possessed significantly lower numbers of T_H_2 (*p* < 0.0001) and Treg cells (*p* < 0.05) and a higher number of T_H_17 cells (*p* < 0.01) compared to the asthmatic mice (Figure 6-c). Similar outcomes were observed when the percentages of each subtype were compared; with the addition that the percentage of antigen-experienced T_H_1 cells in CuO NP-exposed asthmatic mice became significantly lower (*p* < 0.05) (Figure S7-c). CuO NP-exposed asthmatic mice demonstrated a significantly higher number of T-bet^+^ Treg cells (*p* < 0.001) with a significantly lower number of Treg cells expressing GATA-3 compared to asthmatic mice (*p* < 0.001) (Figure 6-d). BAL fluid from CuO NP-exposed asthmatic mice had significantly lower levels of IL-4 (*p* < 0.01) and IL-12(p70) (*p* < 0.05) than asthmatic mice (Figure 6-e). No significant differences occurred in HDM-specific immunoglobulin levels of all types when sera from CuO NP-exposed asthmatic mice and asthmatic mice were compared (Figure 6-f). Lung histopathology of CuO NP-exposed asthmatic mice showed no obvious differences in any parameters compared to those of the asthmatic mice (Figure 6-g). CuO NP exposure to asthmatic mice caused no significant changes in AHR compared to the asthmatic model (Figure 6-h).

#### 2.4.3 CuO NP Exposure to AIT-Treated Asthmatic Mice

**Figure 7:**
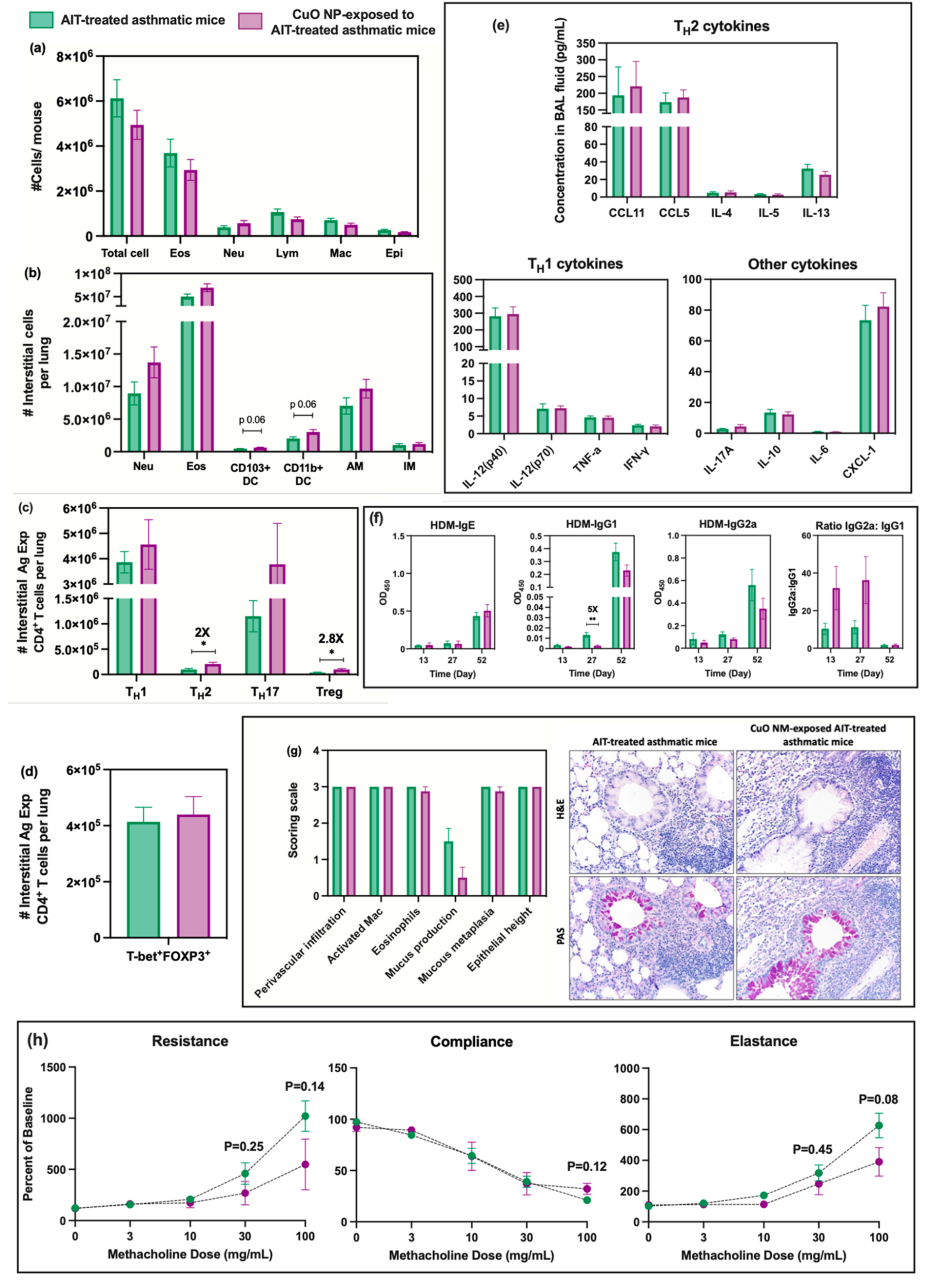
Assessment of immunomodulatory effects of CuO NP inhalation exposure to AIT-treated asthmatic mice (comparison e): (a) cellular analysis of BAL fluid including total cell number, as well as the number of eosinophils (Eos), neutrophils (Neu), lymphocytes (Lym), macrophages (Mac), and epithelial cells (Epi); (b) numbers of indicated interstitial cells including neutrophils (Neu), eosinophils (Eos), CD103^+^ dendritic cells (DCs), CD11b^+^ DCs, alveolar macrophages (AM), and interstitial macrophages (IM) in lung tissue homogenates; (c) numbers of interstitial antigen-experienced CD4^+^ T cells including T_H_1, T_H_2, T_H_17, and Treg cells in lung tissue homogenates; (d) number of interstitial antigen-experienced Treg cells expressing either T-bet or GATA3; (e) cytokines/chemokines from BAL fluid measured by Bio-Plex ProTM mouse cytokine 23-plex assay; (f) serum HDM-specific immunoglobulin (IgE, IgG_1_, IgG_2a_) levels and ratio of IgG_2a_:IgG_1_ measured using an indirect ELISA technique at days 13, 27, and 52; (g) representative micrographs of lung sections from each experimental group after H&E staining (upper row) or Periodic Acid-Schiff (PAS, lower row) staining (magnification 20x). Bar graphs show the scoring scale (0, within the scope of normal; 1, rare, but detectable change; 2, mild in distribution/severity; 3, moderate in distribution/severity; 4, severe in distribution/severity) for 6 parameters including increases in the number of activated macrophages, eosinophils, perivascular infiltration (infiltration of lymphocytes around vessels), mucus production, mucous metaplasia, and epithelial height; (h) pulmonary mechanics measurements of mice to assess AHR. Resistance, compliance, and elastance were measured after the inhalation challenge to increasinge concentrations of methacholine (0, 3, 10, 30, and 100 mg/mL). Statistical analysis was performed using the unequal variance unpaired *t* test (Welch *t* test). Data are shown as mean ± SE (*n* = 6). ****p < 0.0001, ***p < 0.001, **p < 0.01, *p < 0.05.

**Figure 8:**
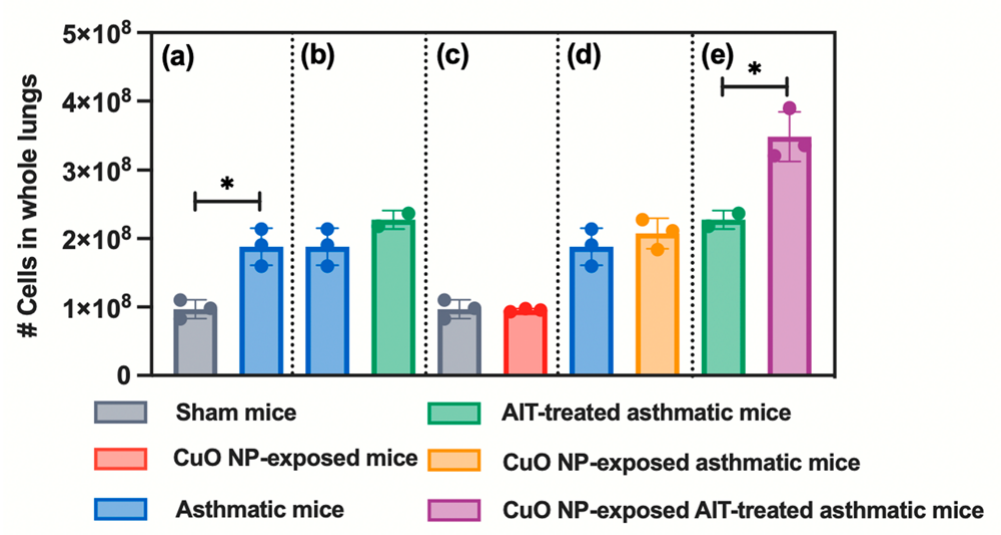
Total cell counts in lung homogenates from indicated treatment groups measured using a Moxi Go II flow cytometer. The total cell count included all cell types in the lung tissue except red blood cells. The following treatment groups were compared statistically: (a) sham mice and asthmatic mice; (b) asthmatic mice and AIT-treated asthmatic mice; (c) sham mice and CuO NP-exposed mice; (d) asthmatic mice and CuO NP-exposed asthmatic mice; (e) AIT-treated asthmatic mice and CuO NP-exposed AIT-treated asthmatic mice. Statistical analysis was performed using the unequal variance unpaired *t* test (Welch *t* test). Data are shown as mean ± SE (*n* = 3). *p < 0.05.

Cellular analysis of BAL fluid from AIT-treated asthmatic mice versus CuO NP-exposed AIT-treated asthmatic mice showed no significant differences in any cell types (Figure 7-a). CuO NP exposure to AIT-treated asthmatic mice caused a significant increase in overall total cell numbers in the lung homogenates (Figure 8) compared to the AIT-treated asthmatic mice; however, there were no significant differences in the numbers and percentages of each cell type examined in lung homogenates (Figure 7-b, Figure S8-b). CuO NP-exposed AIT-treated asthmatic mice showed significantly higher numbers of interstitial antigen-experienced T_H_2 (*p* < 0.05) and Treg cells (*p* < 0.05) than the AIT-treated asthmatic mice, but there was no difference in the levels of Treg cells coexpressing the other examined transcription factors (i.e., Tbet or GATA3) in lung homogenates compared to the AIT-treated asthmatic mice (Figure 7-c,d). The number of Treg cells expressing GATA3^+^ in both tested groups was almost zero; therefore, it is not shown in Figure 7-d. The levels of cytokines in the BAL fluid of CuO NP-exposed AIT-treated asthmatic mice exhibited no differences compared with the levels of cytokines found in the BAL fluid of AIT-treated asthmatic mice (Figure 7-e). The sera from CuO NP-exposed AIT-treated asthmatic mice had significantly lower HDM-specific IgG_1_ levels compared to sera from AIT-treated asthmatic mice on day 27 (*p* < 0.01, Figure 7-f). The effect of CuO NP exposure on AIT-treated asthmatic mice showed a trend toward decreased elastance (*p* = 0.08;100 mg/mL methacholine) compared to AIT-treated asthmatic mice (Figure 7-h).

## 3 Discussion

In our previous studies, the potential immunotoxicity of CuO NP exposure to healthy mice^16^ and pregnant mice (representing a T_H_2 phenotype)^9^ was revealed through the promotion of profound pulmonary inflammation. In addition, we previously found that total Cu levels were significantly elevated in whole blood, indicating that inhaled Cu could be translocated into the bloodstream and that there were differences in gene expression changes related to T_H_1/T_H_2 responses in spleens when healthy and pregnant mice were compared. The goal of this study was to evaluate the effect of CuO NP exposure on innate and adaptive immunity in healthy, house dust mite (HDM) asthmatic or allergen immunotherapy (AIT)-treated asthmatic mouse models. The results revealed that healthy mice exposed to CuO NPs had a significant reduction in T_H_1 cells and T_H_2 cells, and an increase in T-bet^+^ Treg cells, indicating that CuO NP exposure might diminish T_H_2 immunological responses and suppress T_H_1 responses even 40 days after the exposure period had ended. Similar effects were seen in asthmatic mice; however, asthmatic mice receiving AIT exhibited an increase in T_H_2 cells when exposed to CuO NP inhalation. A summary of the results, with significant differences between the investigated models, is shown in Table 1.

**Table 1:** Summary of Results with Significant Differences between Investigated Models

Comparison model	#Cells in BAL	#Interstitial cells	#Interstitial Ag Exp CD4^+^ T cell	Cytokines	Immunoglobulin	Histopathology	Pulmonary mechanic
Asthmatic compared to sham	↑Total cells (13x)	↑Neu (2.6x)	↑T_H_1 (6x)	↑CCL11 (5.2x)	↑IgG1 (36x)	↑Perivascular infiltration	↑Resistance
	↑Eos (450x)	↑Eos (280x)	↑T_H_2 (177x)	↑CCL5 (18x)	↑IgG2a (5x) (Day 52)	↑Activated Mac	↓Compliance
	↑Lym (52x)	↑CD103^+^ DC (2.4x)	↑T_H_17 (26x)	↑IL-4 (24x)		↑Eosinophils	↑Elastance
	↑Mac (2.3x)	↑CD11b^+^ DC (13x)	↑Treg (15x)	↑IL-5 (29x)		↑Mucus production	
	↑Epi (9.3x)	↑AM (6.9x)	↑T-bet^+^ Foxp3^+^ Tregs (19x)	↑IL-13 (2.6x)		↑Epithelial height	
		↑IM (5.3x)	↑GATA3^+^ Foxp3^+^ Tregs (19x)	↑IL-12(p40) (3.2x)			
				↓IL-12(p70) (1.5x)			
				↓IFN-γ (1.4x)			
				↑IL-6 (2.6x)			
				↑CXCL-1 (2.2x)			
AIT-treated asthmatic compared to Asthmatic mice	↑Neu (2.8x)	↑Neu (5.1x)	↑T_H_1 (18x)	↑CCL5 (2.7x)	↑IgG1 (3x)	No difference observed	No difference observed
		↑CD103^+^ DC (1.9x)	↓T_H_2 (10x)	↑IL-12(p40) (2.8x)	↑IgG2a (14x)		
		↑CD11b^+^ DC (1.6x)	↑T_H_17 (17x)	↑IL-17A (2.2x)	↑IgG2a:IgG1 (3x) (Day 52)		
		↑AM (2.6x)	↓Treg (8.5x)				
		↑IM (3.4x)	↑T-bet^+^ Foxp3^+^ Tregs (44x)				
			↓GATA3^+^ Foxp3^+^ Tregs				
CuO NP-exposed mice compared to sham	↓Eos	↑Neu (2x)	↓T_H_1 (4x)	No difference observed	No difference observed	No difference observed	No difference observed
		↓CD11b^+^ DC (3.2x)	↓T_H_2 (3x)				
		↓AM (1.9x)	↑T-bet^+^ Foxp3^+^ Tregs (29x)				
CuO NP-exposed asthmatic compared to asthmatic mice	No difference observed	↑CD103^+^ DC (1.4x)	↓T_H_2 (3x)	↓IL-4 (2.5x)	No difference observed	No difference observed	No difference observed
		↑AM (2.1x)	↑T_H_17 (2.4x)	↓IL-12(p70) (1.6x)			
		↑IM (4.8x)	↓Treg (1.2x)				
			↑T-bet^+^ Foxp3^+^ Tregs (26x)				
			↓GATA3^+^ Foxp3^+^ Tregs (2x)				
CuO NP-exposed AIT-treated asthmatic compared to AIT-treated asthmatic mice	No difference observed	No difference observed	↑T_H_2 (2x)	No difference observed	↓IgG1 (5x) (Days 27)	No difference observed	No difference observed
			↑Treg (2.8x)				

The levels of CD4^+^ T cell subsets with single expression of transcription factors such as Tbet, GATA3, RORgt, and Foxp3 were determined, indicating T_H_1, T_H_2, T_H_17, and Treg cells, respectively. We also monitored the expression of T-bet^+^ and GATA3^+^ by Treg cells because of the plasticity of Treg cells. CD4^+^ T cells can exhibit phenotypic plasticity, allowing them to acquire distinct functions to target certain pathogens as well as reprogram their phenotypes to functionally adapt to changing circumstances.^17^ The plasticity of Treg cells has been demonstrated through their ability to express each of the transcription factors that define each of the T helper cell subsets in response to environmental cues.^18,19^ This expression can be a transient state prior to reprogramming into specific effector CD4^+^ T cells.^17^ In people with food allergies, elevation in IL-4R signaling in Treg cells impaired the capacity of Treg cells to suppress mast cell activation and expansion, which in turn drove reprogramming of Treg cells into T_H_2-like cells.^20^ Our study focused on the expression of either T-bet or GATA-3 by Treg cells (i.e., CD4^+^ Foxp3^+^) because T-bet and GATA-3 are major regulators of T_H_1 and T_H_2 CD4^+^ T cells, respectively.

We confirmed that the HDM-induced asthmatic model employed in our studies was successful as demonstrated by the elevation of eosinophils in BAL fluid and lung homogenates, increased mucus production into the airways, increased airway resistance and elastance and decreased compliance after methacholine challenge, and strong T_H_2-skewed immune responses (cells and cytokines), which are all hallmarks of allergic asthma (Figure 3).^21−23^ In addition, we observed increases in the percentage and number of T_H_17 cells, which are known to be involved in moderate to severe and steroid-insensitive asthma,^24−26^ indicating that the generated asthmatic model caused a high degree of airway inflammation.

Comparing asthmatic versus AIT-treated asthmatic mice; cellular analysis of BAL fluid and lung tissue homogenates from AIT-treated asthmatic mice showed that AIT did not abrogate eosinophilia (Figure 4-a,b) or decrease serum specific-IgE levels (Figure 4-f), which did not align with our expectations. In addition, significant increases in the numbers of neutrophils in BAL fluid and lung homogenates of AIT-treated asthmatic mice (versus asthmatic mice) indicated lung inflammation (Figure 4-a,b).^27,28^ This could be because our asthmatic model causes strong airway inflammation as evidenced by both eosinophil and neutrophil infiltration, and therefore the AIT treatment was insufficient for mitigating the lung inflammation. However, AIT treatment of asthmatic mice led to a decrease in the percentage (as opposed to number) of eosinophils in lung homogenates (Figure S5-b). In addition, we found that the AIT-treated asthmatic mice showed a significant rise in the number and percentage of interstitial T_H_1 cells (*p* < 0.0001), with a decrease in the number and percentage of interstitial T_H_2 cells (*p* < 0.0001) compared to the asthmatic mice, indicating that the vaccine can minimize T_H_2 immune responses and promote T_H_1 immune responses (Figure 4-c, Figure S5-c). The AIT-treated asthmatic mice exhibited an increase in Treg cells expressing T-bet (*p* < 0.01), with decreases in Treg cells expressing GATA-3 (*p* < 0.0001) compared to asthmatic mice (Figure 4-d) which reinforced the T_H_1 and T_H_2 skewing data shown in Figure 4-c. However, there are significantly higher numbers and percentages of T_H_17 cells of the AIT-treated asthmatic mice compared to the asthmatic mice (*p* < 0.05 and *p* < 0.01, respectively) (Figure 4-c, Figure S5-c). This could indicate severe airway inflammation as a consequence of AIT itself and also a robust asthmatic model. The number and percentage of Treg cells in the AIT-treated asthmatic mice were significantly lower than those in the asthmatic mice (*p* < 0.0001, Figure 4-c, Figure S5-c). This could be because Treg cells converted to a transitory stage with simultaneous expression of Tbet and Foxp3 (as shown in higher coexpression of T-bet^+^ Foxp3^+^ CD4^+^ T cells, Figure 4-d) before converting to T_H_1 cells. Alternatively, this observed increase in the number of T-bet^+^ Treg cells could be in response to the increased need to regulate the increased Th1 response. The potential of Treg cells converting to Th1-like cells, coexpressing Foxp3 and Tbet, was described a decade ago^29^ with some Tbet cells showing increased regulatory ability of Th1 cells and others losing suppressive capacity under continuous stimulation. IgG2a:IgG1 ratios in sera indicated that AIT-treated asthmatic mice possessed higher T_H_1 type responses than T_H_2 type responses (Figure 4-f) which concur with T helper cell subset data. Lung histological analysis and pulmonary mechanics from AIT-treated asthmatic mice showed no significant differences compared to the asthmatic mice (Figure 4-g,h). However, the AIT-treated asthmatic group exhibited trends of lower resistance and elastance and slightly increased lung compliance, indicating that AIT treatment may slightly decrease AHR in the asthmatic mouse model. Even though the AIT-treated asthmatic mouse model did not exhibit reduced eosinophilia, there was an increase in the T_H_1 immune response and a decrease in the T_H_2 immune response, suggesting a potential benefit from the treatment. Thus, this model may be useful in modeling T_H_1 immune responses in studies focused on immunomodulatory effects. Nevertheless, the AIT model needs further optimization and efficacy testing if it is to be used as a treatment strategy to decrease asthma phenotype.

The CuO NP-exposed healthy (nonasthmatic) mice had significantly lower numbers and percentages of eosinophils than the sham mice; however, they were still proportionally low (2%) compared to other cell types (Figure 5-a, Figure S6-a) and clinically insignificant. There was no significant difference in number and percentage of neutrophils in BAL fluid from CuO NP-exposed healthy mice (compared to sham mice). In our previous studies, neutrophils were the major cells found in the BAL fluid immediately after completion of exposure to inhaled CuO NPs,^7,11^ but a decline in inflammation, represented by a decreased number of neutrophils at different time points after exposure ended, was seen,^16^ which explains why at 40-days post exposure the number of neutrophils in the BAL fluid was not increased anymore. However, cellular analysis of lung homogenates observed an increase in neutrophil numbers in CuO NP-exposed healthy mice (compared to sham mice) which indicates the presence of residual lung inflammation (*p* < 0.0001, Figure 5-b). Our data showed a significant decrease in AMs in terms of numbers and percentage (*p* < 0.05, Figure 5-b, Figure S6-c). Generally, during acute lung damage, AMs appear to inhibit neutrophil infiltration into the lung, which might provide anti-inflammatory effects.^30^ During inflammation resolution and tissue repair, macrophages are scavenging debris and apoptotic neutrophils and clearing them out; a slight decrease of AMs in lung tissue of CuO NP-exposed mice compared to controls might be an indication of regaining tissue homeostasis. The numbers and percentage of CD11b^+^ DCs in CuO NP-exposed mice were significantly lower than those in the sham mice (*p* < 0.0001 for number and *p* < 0.001 for percentage, Figure 5-b, Figure S6-b). CD11b^+^ DCs have been implicated in the induction of T_H_2 cell immunity;^31,32^ therefore, the lower numbers of CD11b^+^ DCs could potentially contribute to the lower levels of T_H_2 cells observed in CuO NP-exposed mice than the sham mice (*p* < 0.05, Figure 5-c). However, we also found significantly lower levels of T_H_1 cells (number and percentage) in CuO NP-exposed mice than those in the sham mice (*p* < 0.05, Figure 5-c). This decrease in T_H_1 could be related to the significant increase in T-bet^+^ Foxp3^+^ by CD4^+^ T cells in CuO NP-exposed mice compared to sham mice (Figure 5-d, *p* < 0.05), as T-bet^+^ Treg cells have been shown to be better equipped to regulate T_H_1 responses. Alternatively, the increase in coexpression of T-bet and Foxp3 may represent an intermediate stage of development with the potential to differentiate into T_H_1 cells under increased inflammatory conditions.^18^ However, we note that despite a similar increase in T-bet^+^ Treg cells in CuO NP-exposed asthmatic mice, we did not observe an increase in T_H_1 cells (Figure 6). There were no significant differences in cytokine levels from BAL fluid, serum immunoglobulin (IgE, IgG_1_, and IgG_2a_) levels, lung histopathology, or pulmonary mechanics between CuO NP exposed or sham mice (Figure 5).

CuO NP exposure to asthmatic mice revealed no significant changes in terms of cellular infiltration into the BAL fluid when compared to nonexposed asthmatic mice (Figure 6-a (numbers), Figure S7-a (percentage)). In lung homogenates, CuO exposure to asthmatic mice decreased the T_H_2 immunity. In addition, CuO-exposed asthmatic mice showed no increase in T_H_1 cell numbers, while T-bet^+^ Treg cell numbers increased compared to untreated asthmatic mice (Figure S7-c, Figure 6-d). The observed marginal but significant increase in numbers of CD103^+^ DCs in the lung homogenate tissue from CuO NP-exposed asthmatic mice (compared to nonexposed asthmatic mice) may have partially contributed to the decreased numbers of T_H_2 cells in the lungs (Figure 6-b). Cytokine levels in the BAL fluid from CuO NP-exposed asthmatic mice were significantly decreased in IL-4 and IL-12(p70)) compared to the nonexposed asthmatic mice (Figure 6-e, *p* < 0.01 for IL-4 and *p* < 0.05 for IL-12(p70)). There were no significant changes in serum immunoglobulin (IgE, IgG_1_, and IgG_2a_) levels, lung histopathology, and pulmonary mechanics (Figure 6-f–h). Thus, CuO NP exposure prior to inducing an HDM asthmatic condition in mice may cause decreased T_H_2 immune responses. The timing of CuO NP exposure to each model could be the reason why our findings did not correspond to the findings of others,^12,13^ where mice exposed to CuO NP (intranasal instillation) during the challenge phase, or CuO NP aerosol-treated cells derived from asthmatic patients, promoted T_H_2 immunological responses. Park et al. investigated the effect of CuO NP exposure by intranasal instillation during OVA challenge phases and they found that CuO NP aggravated the development of asthma by enhancing AHR.^12^ Mice exposed to TiO_2_ NPs during OVA sensitization showed a decrease in AHR and eosinophilia, while exposure of TiO_2_ NPs during the challenge phase enhanced the airway inflammation and caused loss in body weight.^33^

CuO NP-exposure to AIT-treated asthmatic mice showed no significant differences in any parameters that we monitored when compared to nonexposed AIT-treated asthmatic mice; with the exception of significantly higher T_H_2 and Treg cells and decreased IgG1 levels on days 27 compared to AIT-treated asthmatic mice (Figure 7-c). Overall these results suggest that CuO NP exposure to the AIT-treated asthmatic mouse model may induce T_H_2 immune responses.

Overall, inhalation exposure to CuO NPs tended toward a reduction in T_H_2 responses and increases in T-bet^+^ Treg cells in healthy mice (Figure 5) as well as in asthmatic mice (Figure 6). However, CuO NP-exposed AIT-treated asthmatic mice showed significant increases in T_H_2 cells compared to those of AIT-treated asthmatic mice. As discussed in more detail above, some of the data presented here conflicts with findings from others.^12,13^ Therefore, it would be beneficial to study the effect of CuO NP exposure with these models at different times of CuO NP exposure, for example, during HDM sensitization or during the challenge phase, in order to extend the understanding of the immunomodulatory effects of CuO NPs.

On the other hand, CuO in a bulk form as well as in the form of NP has been shown to have antimicrobial, antifungal, and even acaricidal effects,^34^ and thus it is possible that there might be some effects of CuO NP or Cu ions on the potency of HDM allergens. Nanomaterials interact with various biomolecules when they come in contact, resulting in the formation of “protein coronae” which defines the biological identity and may influence immune responses to these materials^35,36^ and thus further investigation of interactions between CuO NPs, allergens and other proteins in various biological environments (lung lining fluid or serum proteins) may also help to explain different immunomodulatory effects of NPs. The first dose of AIT was administered at the same time that CuO NPs were introduced. From our previous studies tracking Cu concentration in the blood and tissues at several time points throughout and after CuO NP inhalation exposure,^16^ we found that Cu concentrations in blood were increased from day 3 and stayed elevated at the time of the second immunization and at least first HDM sensitization. We have not analyzed the content of Cu in the lungs during HDM challenge (days 42–51); however, it is possible that there were still some residual Cu ions present that may have influenced the potency of the HDM allergen. However, these studies were designed with the focus on the effect of CuO NPs on T_H_1 and T_H_2 dominated responses rather than interaction of CuO NP with allergens.

## 4 Conclusion

Prior to evaluating immunomodulatory effects of CuO NP inhalation exposure, an asthmatic mouse model and an AIT-treated asthmatic mouse model were developed to be used as tools to evaluate the effect of CuO NPs on T_H_2-dominated and T_H_1-dominated immune responses, respectively. Asthmatic mice demonstrated T_H_2-dominated immune responses and pathological asthmatic features such as increases in eosinophil levels and increased mucus production and perivascular infiltration. On the other hand, AIT-treated asthmatic mice demonstrated T_H_1-dominated immune responses and decreases in T_H_2 cell levels.

In healthy mice, CuO NP exposure caused a decrease in T_H_1 and T_H_2 cells and increases in T-bet^+^ Treg cells, which have been shown to be better equipped to regulate T_H_1 responses, indicating that CuO NP exposure could decrease T_H_2 and suppress T_H_1 immune responses even 40 days after last exposure. Similar to CuO NP exposure to asthmatic mice, inhalation exposure to CuO NPs prior to sensitization (asthmatic model) caused a decrease in the T_H_2 immune response and an increase in T-bet^+^ Treg cell levels in the lung homogenates compared to nonexposed asthmatic mice. Conversely, the effect of CuO NP inhalation exposure on AIT-treated asthmatic mice showed an increase in T_H_2 cells and no increase in T-bet^+^ Treg cells.

Overall, the findings partially contradict our hypothesis, where we expected that CuO NP exposure of asthmatic mice would increase T_H_2 immune responses; however, we found a suppression of T_H_2 immunity in this model. However, the effect of CuO NP exposure on AIT-treated asthmatic mice proved our hypothesis of CuO NP exposure increasing the number of T_H_2 cells in the AIT-treated asthmatic mouse model.

## 5 Materials and Methods

### 5.1 Fabrication and Characterization of a Prophylactic Vaccine against HDM-Induced Airway Inflammation

The HDM vaccine contained 2 primary components: 50 μg of class B CpG ODN 1826 (Integrated DNA technologies, Coralville, IA)-loaded poly(lactide-*co*-glycolide) PLGA (Resomer RG 530, Evonik, Germany) NPs and 100 μg of purified Der p1 and Der p 2 (80:20) (lot numbers: 02.01.71 and 02.01.72, respectively, CiteQ Biologics, Groningen, Netherlands) dispersed in 150 μL of saline (Baxter, Deerfield, IL). CpG-loaded PLGA NPs were fabricated using a double emulsion solvent evaporation method as previously described by Joshi et al. with some modifications (Figure 2a, Method S1).^37^ To characterize CpG NPs, hydrodynamic diameter and zeta potential were measured using a Zetasizer (Zetasizer Nano ZS, Malvern Instrument Ltd., Westborough, MA). The primary particle size and surface morphology of the NPs were determined by scanning electron microscopy (SEM). CpG loading was measured using a Quant-iT Oligreen ssDNA assay kit (ThermoFisher Scientific, Waltham, MA).

### 5.2 Experimental Procedure for Immunization, CuO NP Inhalation Exposure, and HDM-Induced Airway Inflammation

Female BALB/c mice (5 weeks old, Jackson Laboratories, Bar Harbor, ME) were housed and maintained in the University of Iowa animal care facilities (Iowa City, IA) with a 12 h light/dark cycle and acclimatized for 7 days before the start of experiments. All animal protocols were approved by the University of Iowa Institutional Animal Care and Use Committee.

Mice were randomly divided into 7 experimental groups consisting of sentinels, sham-exposed mice, CuO NP-exposed mice, asthmatic mouse model (HDM-exposed mice), AIT-treated asthmatic mouse model, CuO NP-exposed asthmatic mouse model, and CuO NP-exposed AIT-treated asthmatic mouse model. Each group contained 12 mice (except the sentinel, which had only 6 mice for analysis of BAL fluid), which were used to measure pulmonary mechanics and lung histopathology (*n* = 6) and to investigate other measurements (*n* = 6) which included total and differential cell counts from BAL fluid, staining of cells from lung tissue homogenates (and analysis using flow cytometry), and measurement of HDM-specific immunoglobulin in serum.

A schematic of the experimental timeline is shown in Figure 1. Mice were immunized by sc injection with the AIT containing 100 μg of Der p1 and Der p2 and 50 μg of CpG NPs in 150 μL of saline (or only 150 μL of saline in the sham mice and asthmatic mice) on days 0 and 14. Two hours after completing the first immunization, mice were exposed via nose-only inhalation to CuO NP aerosols at 3.5 mg/m^3^ for 4 h/day with exposures determined gravimetrically. Inhalation exposure of CuO NP aerosols was performed daily for a total of 5 days/week for 2 weeks (or HEPA-filtered air in the sham group) as described in Method S2. The primary particle size of CuO NPs determined by transmission electron microscopy was 50.2 ± 11.0 nm. A physicochemical characterization of CuO NPs was performed by the Nanotechnology Health Implications Research (NHIR) consortium, Engineered Nanomaterials Resource and Coordination (ERCC), and reported previously.^15,38^ Aerosol size distribution was assessed by using a scanning mobility particle sizer (SMPS, TSI Inc., Shoreview, MN). Concentrations of Cu in lungs and other tissues during and after inhalation exposure to the same CuO NPs as used in this study were determined and reported in our previous study. Estimated deposited doses of CuO NP aerosol in pulmonary regions in this prior study was 53 μg/mouse lung and Cu concentration in lung tissue determined by ICP-MS was the highest immediately after the last day of 10-day exposure (10 μg/lung), with Cu levels in whole blood increasing until 5 days after the exposure ceased.^16^ On days 28 and 35, mice were sensitized with 100 μg of HDM extracts (lot number: 02.01.85, CiteQ biologics, Groningen, Netherlands) in 100 μL of saline by sc injection (sham mice were injected with 100 μL saline). Each 100 μg of HDM extract contains 459 EU endotoxin. One week later, mice were challenged with 25 μg of HDM extracts in 50 μL of saline via intranasal instillation for 10 consecutive days (days 42–51; sham mice were exposed to 50 μL of saline by intranasal instillation). Each 25 μg of HDM extracts contains 115 EU endotoxin. All treatments were performed while mice were under anesthesia with isoflurane (Akorn, Inc., Lake Forest, IL), except for CuO NP aerosol exposure. On day 52, six mice (per group) were euthanized with an overdose of isoflurane, and then sera, BAL fluid, and lung tissue were collected to measure serum HDM-specific immunoglobulin levels (IgE, IgG_1_, IgG_2a_), cytokines, total cell counts, and the numbers of leukocyte subsets from either BAL fluid or lung homogenate. Measurement of pulmonary mechanics was conducted to assess AHR in the other 6 mice from each group.

This study has 5 main comparisons of interests: (a) sham versus asthmatic mice, (b) asthmatic mice versus AIT-treated asthmatic mice, (c) sham-exposed versus CuO NP-exposed mice, (d) asthmatic mice versus CuO NP-exposed asthmatic mice, and (e) AIT-treated asthmatic mice versus CuO NP-exposed AIT-treated asthmatic mice.

The asthmatic mice were compared to the sham mice (comparison a) to confirm that the HDM exposure regime successfully generated asthmatic conditions, as indicated by increases in eosinophils, T_H_2 cells, serum HDM-specific IgE and IgG_1_ levels, AHR, and lung histopathology changes (i.e., increases in perivascular infiltration, mucus production, and epithelial height).

The AIT-treated asthmatic mice were compared to the asthmatic mice (comparison b) to investigate the efficacy of AIT, which should exhibit increases in T_H_1 cells and serum HDM-specific IgG_2a_, while decreases in eosinophilia, T_H_2 cells, serum HDM-specific IgE and IgG_1_ levels, and AHR were observed.

To observe the effect of CuO NP exposure in a naïve mouse model, we compared the outcomes between the sham mice and CuO NP-exposed mice (comparison c) to determine whether CuO NP exposure still had residual inflammation at the necropsy time point as represented by increased numbers of neutrophils or changes in numbers of macrophages in BAL fluid or lung tissue homogenates.

The asthmatic mice were compared to CuO NP-exposed asthmatic mice (comparison d) to investigate the effect of CuO NP exposure in the asthmatic mouse model. The AIT-treated asthmatic mice were compared to the CuO NP-exposed AIT-treated asthmatic mice (comparison e) to investigate the effect of CuO NP on the efficacy of AIT. The effect of CuO NP exposure on the asthmatic and AIT-treated asthmatic mouse model could result in changes in cell infiltration into the lungs, serum HDM-specific immunoglobulin levels, AHR and lung histopathology. We hypothesized that CuO NP exposure would exacerbate asthmatic conditions and decrease the efficacy of AIT.

### 5.3 Intravascular Staining to Determine Cellular Localization

Cells of the immune system act locally to alleviate, prevent, or exacerbate diseases; therefore, tissue-localized cells must be characterized. To discriminate cells within tissue from cells within vasculature, intravascular staining was performed as previously described.^39^ Briefly, 3 min prior to euthanasia, mice were injected with 1 μg of Brilliant Violet 570 rat antimouse CD45.2 (Clone 104; Biolegend, San Diego, CA), a pan leukocyte marker that is expressed by all leukocytes in BALB/c mice,^40^ in 200 μL of 1X PBS by retro-orbital intravenous injection under anesthesia. Within 3 min of injection, the leukocytes positively stained with the CD45.2 antibodies were considered as cells within the blood, while those negatively stained with the CD45.2 were considered as interstitial cells (tissue-localized cells) and were of interest here. Three minutes after injection, mice were euthanized with an overdose of isoflurane followed by cervical dislocation, thoracotomy, and exsanguination through the heart to collect blood, BAL fluid, and lung tissue.

### 5.4 Cellular Analysis of BAL Fluid

BAL fluid was collected from the right lobes by lung lavage with 1 mL of sterile sodium chloride solution (0.9%, Baxter, Deerfield, IL) 3 times. The collected BAL fluid was centrifuged at 800*g* for 5 min at 4 °C to separate the cellular components and supernatant. The cell pellets were resuspended in 200 μL of Hank’s balanced salt solution (Life Technologies, Grand Island, NY) and the total cell count (which included all cell types) was determined using a Moxi Go II flow cytometer (Orflo technologies, Ketchum, ID). Then, the cells were stained with Protocol Hema 3 Fixative and solutions (Fisher scientific, Waltham, MA) to differentiate the cells into eosinophils, neutrophils, macrophages, and lymphocytes. Both percentages and frequencies of each cell type were reported to account for the cellular distribution (% of total cell count) and recruitment to the airways (total number), respectively.

### 5.5 Preparation of Lung Homogenates and Antibody Staining for Flow Cytometric Analysis

After harvesting the BAL fluid (see section 2.4), the whole lungs were cut into small pieces and incubated for 30 min at 37 °C in 10 mL of digestion media containing 1 mg/mL collagenase D and 0.02 mg/mL DNase (both from Roche, Indianapolis, IN) in complete Iscove’s Modified Dulbecco’s Medium (IMDM, Gibco, Grand Island, NY). After incubation, the lung tissue was dissociated using a GentleMACS dissociator (Miltenyi Biotec, Bergisch Gladbach, Germany) and passed through a 70 μm cell strainer to obtain single-cell suspensions. The resultant cells were counted using a hemocytometer along with trypan blue staining to assess viability.

In this experiment, we performed antibody staining on the prepared single cell suspension from lung homogenates for flow cytometry analysis, using 2 antibody panels: a panel for detecting interstitial CD4^+^ T cell subsets (T cell panel, Table S1) and a panel for detecting interstitial neutrophils, eosinophils, dendritic cells, and macrophages (antigen presenting cell (APC) panel, Table S2).

For T cell panel staining, single cell suspensions (1 × 10^6^ cells/well) were placed in one well of a 96-well round bottomed plate (Corning star, Glendale, AZ) and labeled with live/dead Zombie UV fixable viability dye according to the manufacturer’s instructions (BioLegend) for 15 min at 4 °C in dark conditions. Then cells were blocked with 2% v/v rat serum and 2% v/v hamster serum (Jackson ImmunoResearch Laboratories, West Grove, PA) in cell staining buffer (BioLegend) for 30 min at 4 °C. Cells were stained with 100 μL of fluorochrome conjugated surface marker antibodies as shown in Table S1 diluted in cell staining buffer and incubated with cells (45 min at 4 °C, protected from light) to identify antigen-experienced (CD44^hi^ CD11a^hi^) T cells. After washing with 200 μL of cell staining buffer once (centrifuged at 500*g* for 5 min), cells were stained with fluorescence conjugated antibodies specific for transcription factors including T-bet, GATA 3, RORgt, and Foxp3 in order to differentiate between T_H_1 (Tbet single positive), T_H_2 (GATA3 single positive), T_H_17 (RORgT single positive), and Treg (Foxp3 single positive) cells (Table S1) using a protocol following the manufacturer’s instructions for staining with Foxp3 transcription factor-specific antibodies (Thermofisher, Method S3). Data were acquired on a Cytek Aurora (Cytek Biosciences, Fremont, CA) in the University of Iowa Flow Cytometry Facility and analyzed using FlowJo software (Tree Star, Ashland, OR). The gating strategy used to identify interstitial CD4^+^ T cell subsets including T_H_1, T_H_2, T_H_17, and Treg cells in lung homogenates as well as Treg cells expressing Tbet or GATA3 was identified as shown in Figure S2. The data were presented as the numbers and percentages of each subset of antigen-experiment CD4^+^ T cells.

To stain lung cells (after homogenization) with specific antibodies from the APC panel (to detect interstitial myeloid populations), single-cell suspensions (1 × 10^6^ cells/well) were stained using the same initial steps as the protocol employed for the T cell panel until the surface staining step where the APC panel of fluorochrome-conjugated antibodies was used (as shown in Table S2) instead of the T cell panel. Then, the cells were washed twice with 200 μL of cell staining buffer and fixed with 200 μL of 1X BD FACS Lysing solution (diluted in water) for 10 min at room temperature (RT) in the dark. Then the cells were washed twice and resuspended in 200 μL of cell staining buffer. The gating strategy used to identify interstitial cell populations in the lung homogenates including neutrophils, eosinophils, AMs, IMs, CD103^+^ DCs, and CD11b^+^ DCs is shown in Figure S3. The data are presented in the numbers and percentages of CD45.2 iv Ab^–^ cells. The gating strategy for the APC panel is shown in Figure S3.

### 5.6 Measurement of Serum HDM-Specific Immunoglobulin Levels

Mouse serum was collected on days 13, 27, and 52 post first immunization to measure serum levels of HDM-specific IgE, IgG_1_, and IgG_2a_ using an indirect ELISA method described in Method S4. Serum samples were collected on day 13 post first immunization to determine the effect of first immunization in AIT-treated asthmatic mouse group; the CuO NP-exposure effect on CuO NP-exposed mice; or the combined effect of the first immunization and CuO NP exposure for CuO NP-exposed AIT-treated asthmatic mice. Serum samples were collected on day 27 to assess the effect of the second immunization in AIT-treated asthmatic mice; or the effect of CuO NP exposure (15 days post CuO NP exposure); or the combined effect of CuO NP exposure plus 2 immunizations. To measure the overall effect, mouse sera were collected at the necropsy time-point (Day 52 post first immunization).

### 5.7 Measurement of Cytokines/Chemokines in BAL Fluid

The levels of the following cytokines/chemokines in BAL fluid were simultaneously determined using the Bio-Plex Pro mouse cytokine 23-plex assay (Bio-Rad laboratories, Hercules, CA) according to the manufacturer’s protocol and measured using the Luminex 200 system (Bio-Rad): CCL11, G-CSF, GM-CSF, IFN-γ, IL-1α, IL-1β, IL-2, IL-3, IL-4, IL-5, IL-6, IL-9, IL-10, IL-12 (p40), IL-12 (p70), IL-13, IL-17A, CXCL-1, MCP-, MIP-1α, MIP-1β, CCL5, and TNF-α. The levels of T_H_1 (i.e., IL-12 (p40), IL-12(p70), TNF-α, IFN-γ), T_H_2 (i.e., IL-4, IL-5, IL-13, CCL11, CCL5), T_H_17 (i.e., IL17A), and Treg cell (i.e., IL-10) associated cytokines present in the BAL fluid were measured to assess the type of immune response promoted in response to the various treatments and conditions.

### 5.8 Pulmonary Mechanics

Pulmonary mechanics were performed to assess AHR on day 52 (1 day after 10th challenge) in all experimental groups (*n* = 6), except the sentinel. Mice were anesthetized with 90 mg/kg of pentobarbital sodium (Oak Pharmaceuticals, Inc. Deerfield, IL, USA) by intraperitoneal injection, and a tracheotomy was performed using a tracheal cannula with a Luer adapter (Outside diameter 1.3 mm, length 20 mm, Harvard Apparatus, Holliston, MA, USA). Mice were then connected to a small animal ventilator (FlexiVent, SCIREQ, Montreal, QC, Canada), set at a frequency of 150 breaths/min, a tidal volume of 10 mL/kg and a positive end-expiratory pressure of 2–3 cm H_2_O. Mice were challenged with increasing concentrations of methacholine chloride (ICN Biomedicals, Inc. Solon, OH) aerosol including 3, 10, 30, and 100 mg/mL, which were generated with an in-line nebulizer (10 s) directly through the canulated trachea. The data were presented as dynamic resistance (R*,* the level of constriction in the lung) and dynamic compliance (*C*, the ease with which the lung can be stretched). Dynamic elastance (*E*, the inverse of the compliance, *E* = 1/*C*) was calculated by flexiVent software (version 8, service pack 4.0). These measurements *R*-*E* represent parameters of the whole respiratory system (airways, lung, and chest wall). After the measurements, mice were disconnected from the ventilator and euthanized, and blood and tissues were collected.

### 5.9 Lung Histopathology

Lung histological analysis was carried out to identify the pathological features of airway inflammation and allergic asthma using hematoxylin and eosin (H&E) and Periodic Acid-Schiff (PAS) staining. Lungs were collected from the mice after pulmonary mechanics measurements. The right lobes of the lung were perfused with 10% buffered formalin (Fisher Scientific, Kalamazoo, MI) through the cannulated trachea and then stored in 10% buffered formalin until further processing. Tissues were subsequently paraffin-embedded, sectioned at 5 μm thickness, and stained with H&E and PAS. Lung tissues were evaluated for key histopathologic changes including increases in the number of activated macrophages, eosinophils, perivascular infiltration (infiltration of lymphocytes around vessels), mucus production, mucous metaplasia, and epithelial height.

### 5.10 Statistical Analysis

The data for comparison between 2 groups were statistically analyzed using unequal variance unpaired T Test (Welch *t* test) using GraphPad Prism (GraphPad software, San Diego, CA). The results from pulmonary mechanics data were not normally distributed; thus the unpaired Mann–Whitney rank test was used to test differences between two groups of interest. A *p*-value less than 0.05 was considered statistically significant. Statistical probability, *p* values in plots are expressed as follows: ****p < 0.0001, ***p < 0.001, **p < 0.01, and *p < 0.05. Data are expressed as mean ± standard error (SE).

## Supporting Information Available

Preparation of CpG ODN-loaded PLGA NPs; preparation and characterization of CuO NP aerosol; transcription factors staining on antigen-experienced T cells from lung tissue homogenate; measurement of serum HDM-specific immunoglobulin; particle size distribution of CuO NP aerosol measured by scanning mobility particle sizer (SMPS); gating strategy used to identify interstitial single expression CD4^+^ T cell subsets and coexpression of T-bet^+^ Foxp^+^ and GATA3^+^ Foxp3^+^ CD4^+^ T cells; gating strategy used to identify extravascular antigen presenting cell populations; percentages of cells in BAL and in lung homogenates; list of surface staining antibodies and transcription factor staining antibodies for discrimination of antigen-experienced CD4^+^ T cell subsets; list of surface staining antibodies and transcription factor staining antibodies for discrimination neutrophils, eosinophils, macrophages, and dendritic cells (PDF)
