# Supplementary material for: Immunomodulatory Effects of Subacute Inhalation Exposure to Copper Oxide Nanoparticles in House Dust Mite-Induced Asthma
Source: ACS Nano. 2023 Jul 18;17(15):14586–603. doi: 10.1021/acsnano.3c01668 (PMC10416562; doi:10.1021/acsnano.3c01668)
Supplement: Supplementary file 1 — nn3c01668_si_001.pdf [file nn3c01668_si_001.pdf]

Immunomodulatory effects of sub-acute inhalation exposure to copper oxide nanoparticles in house dust mite-induced asthma

Sudartip Areecheewakul[1, #], *Andrea Adamcakova-Dodd*[2*], *Zeb R. Zacharias*[3], *Xuefang Jing*[2], *David K. Meyerholz*[4], *Kevin L. Legge*[3,5], *Jon C.D. Houtman*[5], *Patrick T. O'Shaughnessy*[2], *Peter S. Thorne*[2, 6*], *Aliasger K. Salem*[1*]

[1]Department of Pharmaceutical Sciences and Experimental Therapeutics, University of Iowa, Iowa City, IA 52242, USA

[2]Department of Occupational and Environmental Health, University of Iowa, Iowa City, IA 52242, USA

[3]Interdisciplinary Immunology Graduate Program, Department of Pathology, University of Iowa, Iowa City, IA 52242, USA

[4]Department of Pathology, University of Iowa, Iowa City, IA 52242, USA

[5]Department of Microbiology and Immunology, Carver College of Medicine, University of Iowa, Iowa City, IA 52242, USA

[6]Human Toxicology Program, University of Iowa, Iowa City, IA 52242, USA

Supporting information

Method S1. Preparation of CpG ODN-loaded PLGA NPs

4 μg of CpG ODN (Integrated DNA technologies, Coralville, IA) was dissolved in 100 μL 1% poly (vinyl alcohol) (PVA; MW 67,000, Sigma, Allentown, PA) in distilled water. The primary emulsion was formed by sonicating the CpG solution in 200 μg PLGA 50:50 (Resomer RG 530, Evonik, Germany) dissolved in 2 mL dichloromethane (DCM) at 60% amplitude for 60 s. The primary emulsion was added to 8 mL 2.5 % PVA in 0.5X phosphate buffered saline (PBS) (Gibco) with sonication for 60 s to form a secondary emulsion. The secondary emulsion was then added to 22 mL 2.5% PVA in 0.5X PBS and stirred for 2 h under a fume hood to evaporate DCM. Large particles were removed by centrifugation (Eppendorf 5804 R, Westbury, NY) at 300 xg for 5 min, and the remaining particles were collected at 15,000 xg and washed with distilled water 2 times. The particles were lyophilized using FreeZone 4.5 (Labconco Corporation, Kansas City, MO) for 24 h.

Method S2. Preparation and characterization of CuO NM aerosol

CuO NMs were provided by the Engineered Nanomaterials Resource and Coordination Core (ERCC) with a primary particle size at 50.2 ± 11.0 nm. To preparation of CuO NMs aerosol, dry powder consisting of CuO NPs was suspended in distilled water at 1 mg/mL and sonicated using a cup horn sonicator (QSonica, CT) at 75% amplitude for 5 min. The suspension was transferred to a nebulizer glass jar with a magnetic bar, and placed on a magnetic stirrer to prevent CuO NP sedimentation during inhalation exposure. CuO NP aerosols were generated by a 6-jet Collison nebulizer (BGI Inc., Waltham, MA) supplied with dehumidified and HEPA filtered air.

The generated aerosol was passed through a brass drying column heated at 110°C, a humidity condensation jar, and a particle neutralizer (containing 10 mCi ^{85}Kr source, TSI Inc., Shoreview, MN) prior to entering two nose-only inExpose towers holding 12 mice in each tower. The CuO NP aerosol concentration was monitored during the exposure using a personal DataRAM™ pDR-1500 Aerosol Monitor (ThermoFisher Scientific™, Waltham, MA) to control the concentration at 3.5 mg/m^3. The NP concentration was measured gravimetrically using a Mettler XPE-26 microbalance on a pneumatic vibration isolator table (Newport, VIS IsoStation) in a temperature- and humidity-controlled gravimetrics laboratory. The particle size distribution of the generated aerosol was measured by a scanning mobility particle sizer (SMPS, TSI Inc., Shoreview, MN) and presented as geometric mean (GM) and geometric standard deviation (GSD) (Figure S1).

Method S3. Transcription factors staining on antigen experienced T cells from lung tissue homogenate.

After finished surface staining, cells were fixed and permeabilized with 100 μL 1X fixation/permeabilization buffer overnight at 4°C. Then 100 μL 1X permeabilization buffer was added before centrifuging at 500 xg for 5 min. The cells were washed with 200 uL 1X permeabilization buffer once and were stained with 100 μL transcription factor-specific antibodies, diluted in 1X permeabilization buffer, for 30 min at RT. The cells were then washed twice with 200 μL 1X permeabilization buffer and then resuspended with 100 μL cell staining buffer, and these cells were then ready to measure with flow cytometer. Data were acquired on a Cytek Aurora (Cytek Biosciences, Fremont, CA) and analyzed using FlowJo software (Tree Star, Ashland, OR).

Method S4. Measurement of serum HDM-specific immunoglobulin

First, the wells of a 96-well EIA/RIA clear flat bottom high bind microplate (Corning, Glendale, AZ) were coated with 100 μL of HDM extracts (10 μg/mL for IgG_1 and 20 μg/mL for IgG_{2a} and IgE) diluted in 0.05 M carbonate/bicarbonate buffer (pH 9.6) and incubated overnight at 4° C. The plates were washed 3 times with 150 μL washing buffer (0.05% tween-20 in 1X PBS) and blocked with 100 μL 10% fetal bovine serum (FBS, Sigma-aldrich, St. Louis, MO) in 1X PBS for 1 hour at RT. Serum samples and standards were diluted with 10% FBS in 1X PBS. Serum samples for IgG_1 detection used a series of 10-fold dilutions starting from 1:20; for IgG_{2a} detection a series 2-fold dilutions starting from 1:20 was used: and for IgE detection a series of 2-fold dilutions starting from 1:10 was used. Standard samples (Chondrex, Woodinville, WA) involved serial 2-fold dilutions using different concentration ranges as follows: mouse anti-HDM IgG_1 (100-1.6 ng/mL), mouse anti-HDM IgG_{2a} (50-0.8 ng/mL), and mouse anti-HDM IgE (50-0.8 ng/mL). After 3 washes, 100 μL serum samples and standards were added, and the plates were incubated for 2 hours at RT. After 6 washes, 100 μL goat anti-mouse-IgG_1, IgG_{2a}, or IgE antibody conjugated to HRP (dilution 1:4000 in 10% FBS in 1X PBS) were added and the plates were incubated for 1 hour at RT. The plate was washed 6 times and then 100 μL TMB substrates (BDbiosciences, San Jose, CA) were added. The plates were incubated for 30-45 min at RT in the dark, and then 50 μL 2N H_2SO_4 was added to stop the reaction. The absorbance was measured at 450 nm using SpectraMax M5 plate reader.

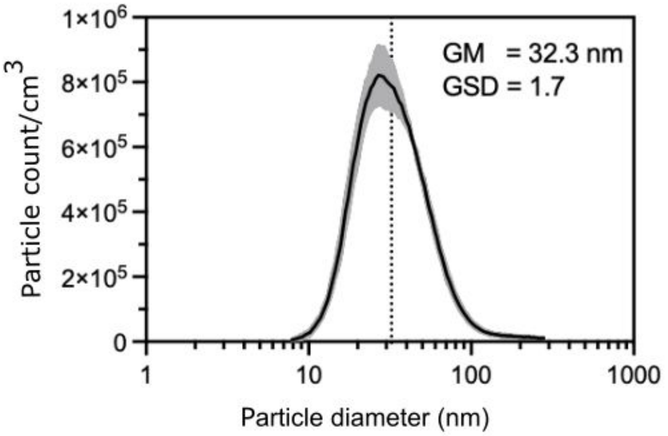

Figure S1. Particle size distribution of CuO NM aerosol measured by scanning mobility particle sizer SMPS.

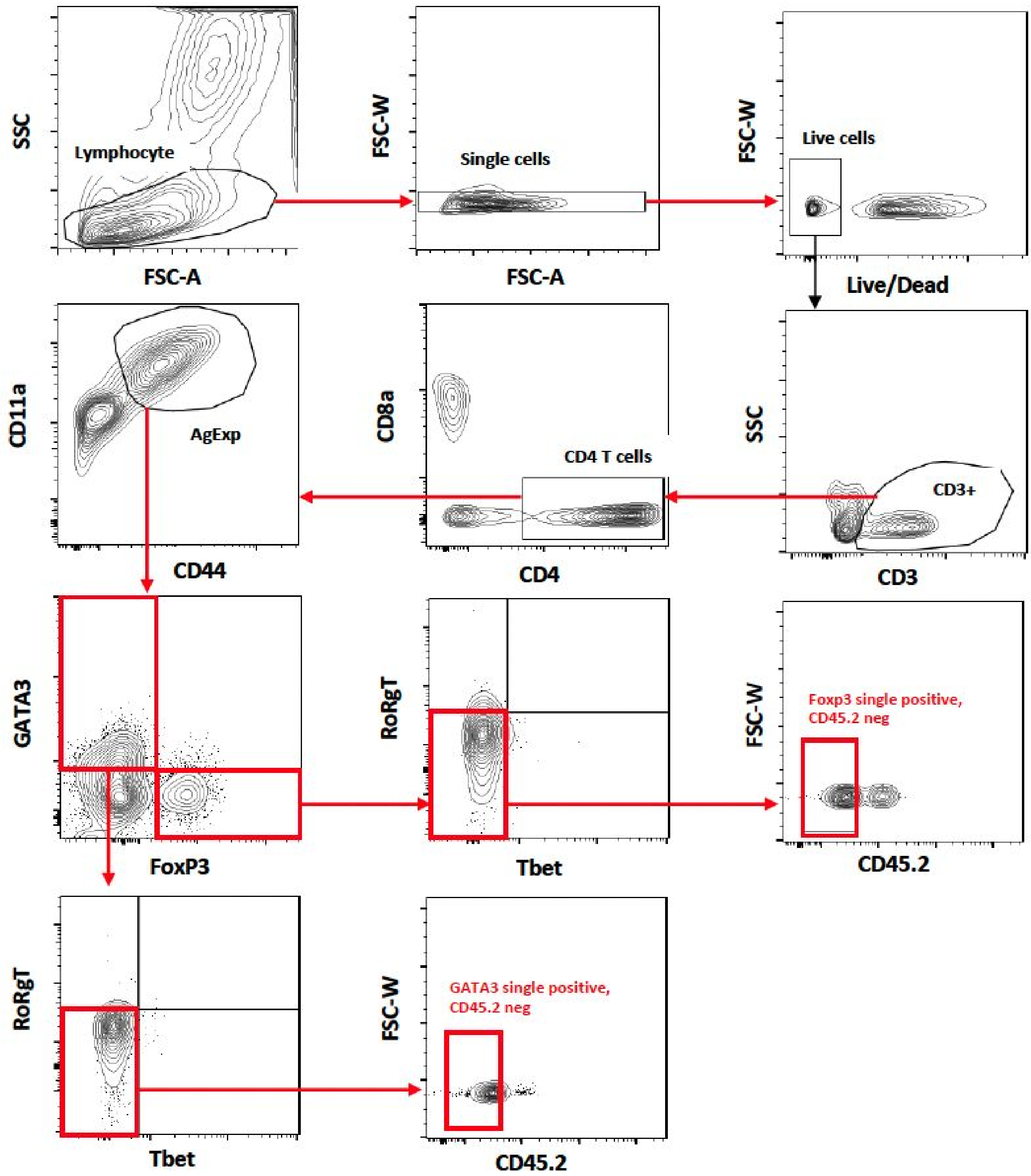
SSC
Lymphocyte
FSC-A
FSC-W
Single cells
FSC-A
FSC-W
Live cells
Live/Dead
CD11a
AgExp
CD44
CD8a
CD4 T cells
CD4
SSC
CD3+
CD3
GATA3
FoxP3
RoRgT
Tbet
FSC-W
Foxp3 single positive,
CD45.2 neg
CD45.2
RoRgT
Tbet
FSC-W
GATA3 single positive,
CD45.2 neg
CD45.2

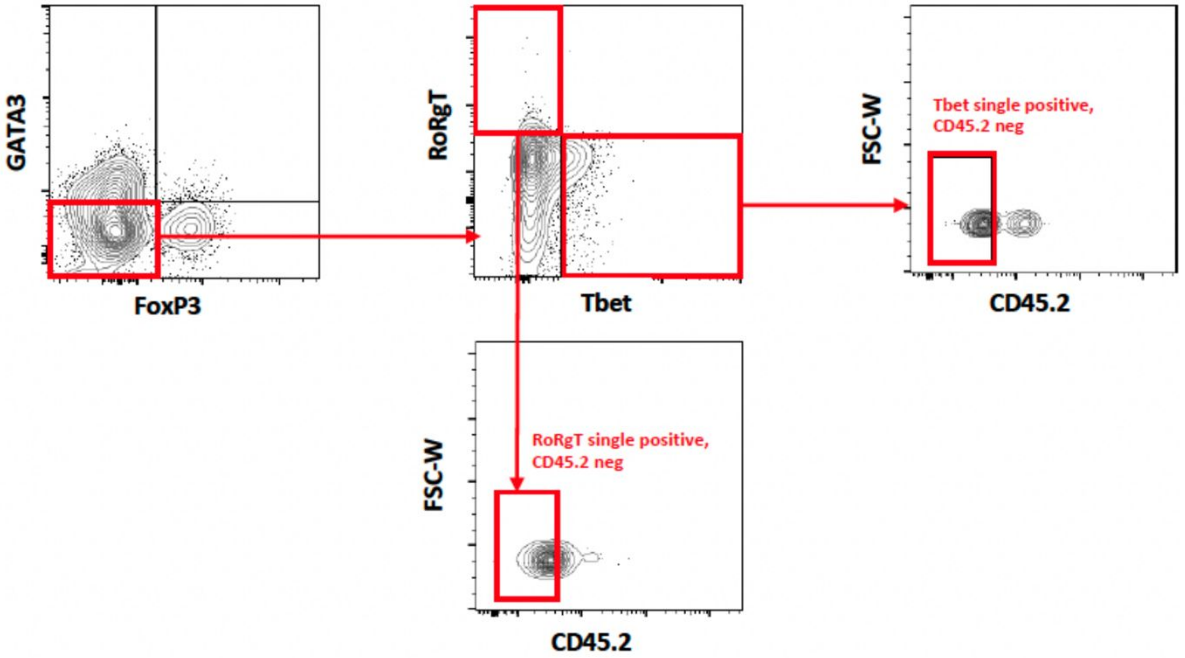

Co-expression: This gating strategy was followed after gating for antigen experience CD4+ Tcells

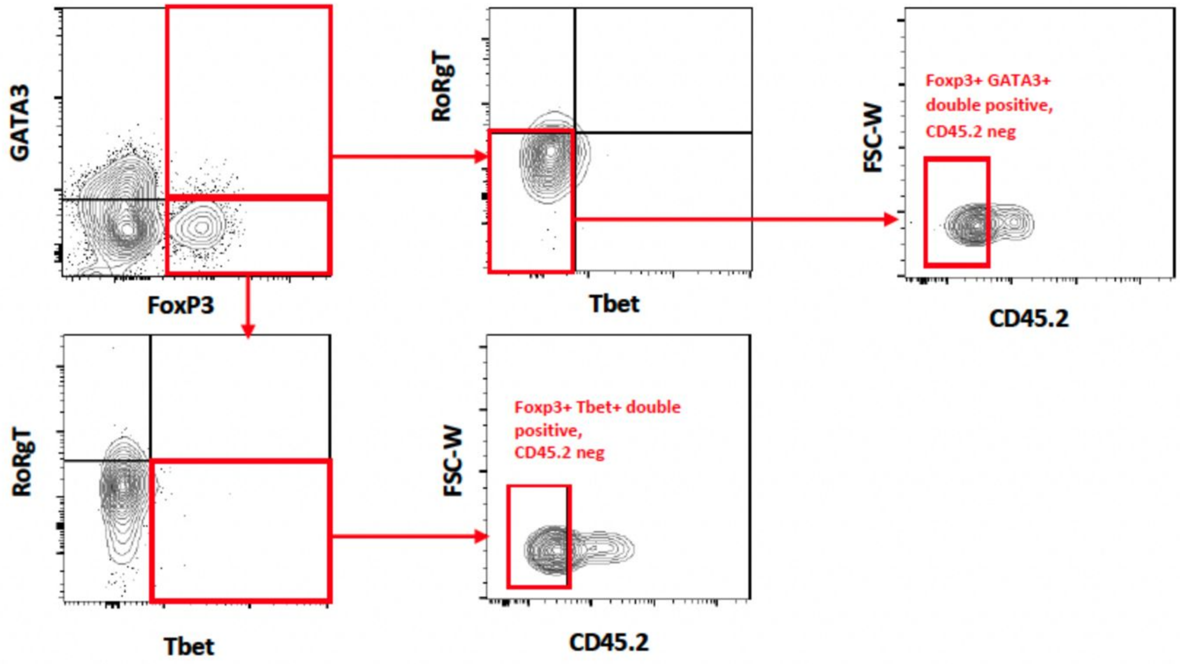

Figure S2. Gating strategy used to identify interstitial single expression $CD4^+$ T cell subsets including T_H1, T_H2, T_H17, and T_{reg} cells in lung tissue homogenates and co-expression of Tbet+Foxp+ and GATA3+foxp3+ $CD4^+$ T cells. The example shown here was from asthmatic mouse lung. Single-cell suspensions were isolated and dissociated from mouse lung and stained with a 12-color flow cytometry panel (Table 5.1). After exclusion of debris, granulocytes, and doublets, dead cells were excluded using live/dead staining. A sequential gating was first used to identify antigen-experienced ($CD44^{hi}$ $CD11a^{hi}$) $CD4^+$ T cell populations using specific markers: $CD3^+$, $CD4^+$, $CD44^{hi}$, and $CD11a^{hi}$. Then, to specifically distinguish them into each subtype of $CD4^+$ T cells, we used transcription factor markers: T-bet for T_H1 cells, GATA-3 for T_H2 cells, RORgt for T_H17 cells, and Foxp3 for T_{reg} cells (The gating was determined based on fluorescence minus one (FMO) sample). Then, we gated for extravascular cells (i.e. the cells that were not stained with the i.v. administered CD45.2 antibody).

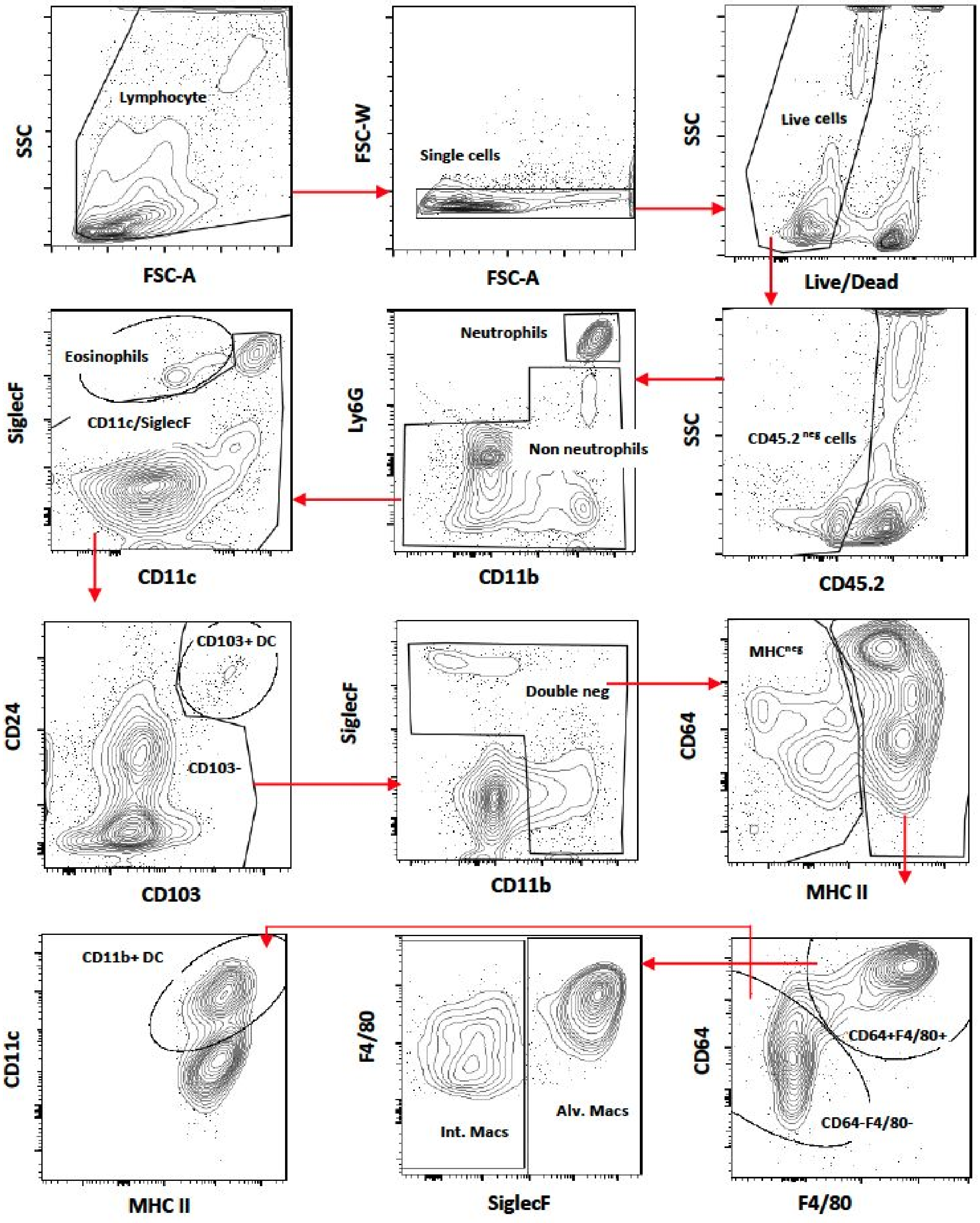
Lymphocyte
SSC
FSC-A
Single cells
FSC-W
FSC-A
Live cells
SSC
Live/Dead
Eosinophils
CD11c/SiglecF
SiglecF
CD11c
Neutrophils
Non neutrophils
Ly6G
CD11b
CD45.2 neg cells
SSC
CD45.2
CD103+ DC
CD103-
CD24
CD103
Double neg
SiglecF
CD11b
MHCneg
CD64
MHC II
CD11b+ DC
CD11c
MHC II
Int. Macs
Alv. Macs
F4/80
SiglecF
CD64+F4/80+
CD64-F4/80-
CD64
F4/80

Figure S3. Gating strategy used to identify extravascular antigen presenting cell populations in the lungs using a 16-color flow cytometry panel (Table 5.2). The debris, doublets, and dead cells were excluded and followed by gating on CD45.2 negatively staining cells to identify extravascular cells. Sequential gating was performed to distinguish populations expressing specific markers: neutrophils ($CD11b^{+}$ $Ly6G^{+}$), eosinophils (Siglec F^{+} $CD11b^{+}$ $CD11C^{-}$), $CD103^{+}$ DCs ($CD103^{+}$ $CD24^{+}$), followed by exclusion of double negative CD11b and Siglec F. Alveolar macrophages (Alv. Macs) were identified as Siglec F^{+} $CD11b^{-}$ $F4/80^{+}$ $CD64^{+}$, while interstitial macrophages (Int. Macs) expressed Siglec F^{-} $MHCII^{+}$ $CD11b^{+}$ $CD64^{+}$ $F4/80^{+}$. $CD11b^{+}$ DCs were identified by the expression of $CD11c^{+}$ MHC II^{+} $CD64^{-}$ $CD24^{+}$ $CD103^{-}$. We performed FMOs for every antibody and used them to gate for the cell population that did not show the distinction between positive and negative stained cells.

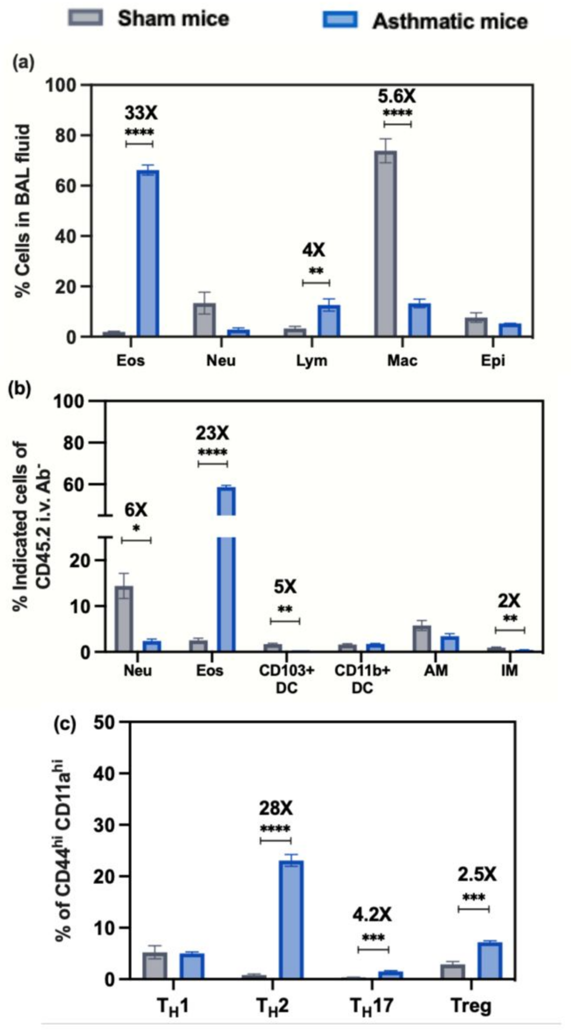

Figure S4. Assessment of asthmatic mice compared to sham mice (comparison a): (a) percentages of cells in BAL fluid including eosinophils, neutrophils, lymphocytes, macrophages, and epithelial cells; (b) Percentages of each indicated subtype from parent cells (*see definition of parent cells in method 2.5*) in the lung tissue homogenates; (c) percentages of $CD44^{hi}$ $CD11a^{hi}$ $CD4^{+}$ T cells in lung tissue homogenates. Statistical analysis was performed using unequal variance unpaired T test (Welch T test). Data were shown as mean ±SE (n=6). ****P<0.0001, ***P<0.001, *P<0.05.

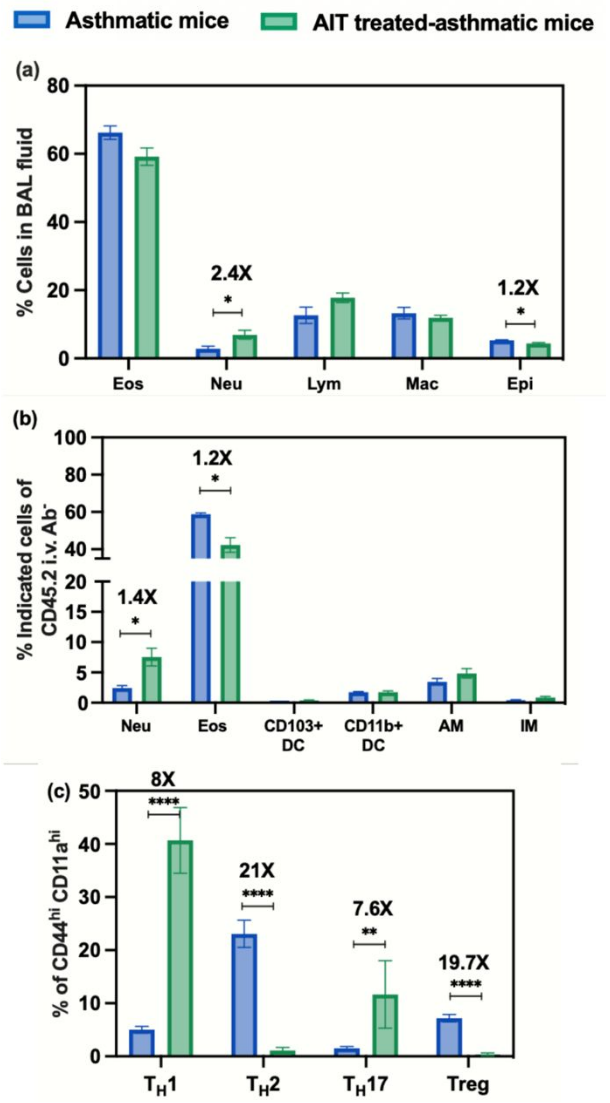

Figure S5. Assessment of AIT-treated asthmatic mice model compared to asthmatic mice (comparison b): (a) percentages of cells in BAL fluid including eosinophils (Eos), neutrophils (Neu), lymphocytes (Lym), macrophages (Mac), and epithelial cells (Epi); (b) Percentages of each indicated subtype from parent cells (*see definition of parent cells in method 2.5*) in the lung tissue homogenates; (c) percentages of $CD44^{hi}$ $CD11a^{hi}$ $CD4^{+}$ T cells in lung tissue homogenates. Statistical analysis was performed using unequal variance unpaired T test (Welch T test). Data are shown as mean ±SE (n=6). ****P<0.0001, ***P<0.001, *P<0.05.

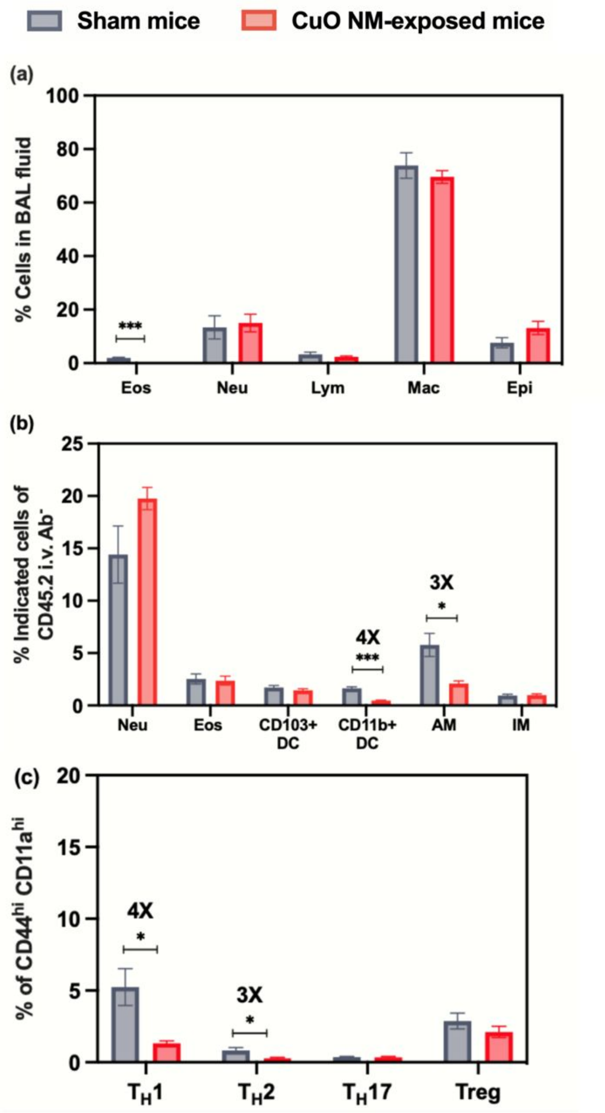

Figure S6. Assessment of immunomodulatory effects of CuO NM inhalation exposure to naive mice (comparison c): (a) percentages of cells in BAL fluid including eosinophils, neutrophils, lymphocytes, macrophages, and epithelial cells; (b) Percentages of each indicated subtype from parent cells (*see definition of parent cells in method 2.5*) in the lung tissue homogenates; (c) percentages of $CD44^{hi}$ $CD11a^{hi}$ $CD4^{+}$ T cells in lung tissue homogenates. Statistical analysis was performed using unequal variance unpaired T test (Welch T test). Data are shown as mean ±SE (n=6). ****P<0.0001, ***P<0.001, *P<0.05.

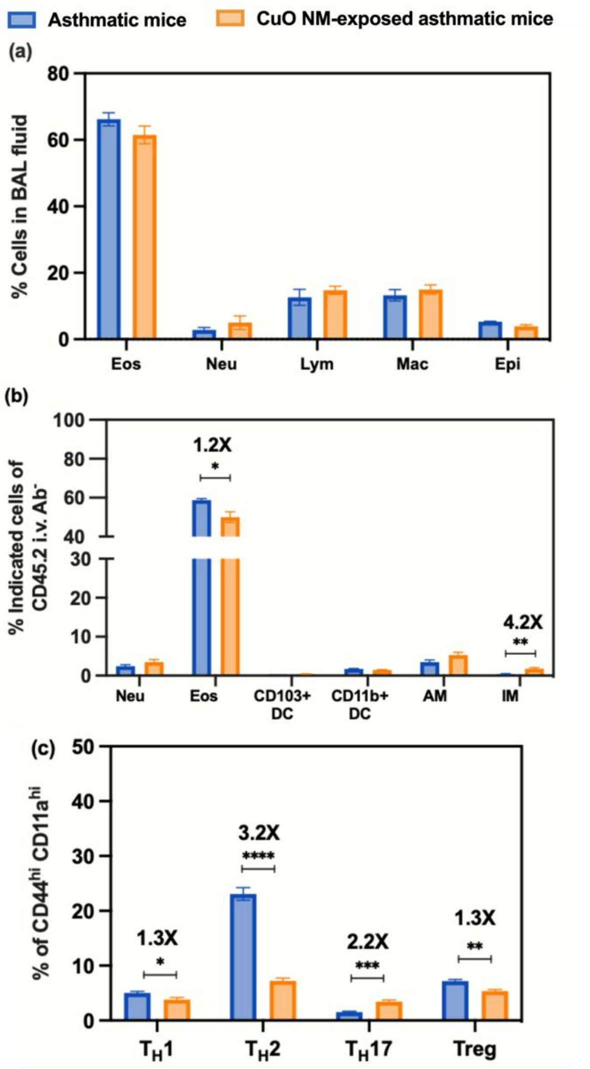

Figure S7. Assessment of immunomodulatory effects of CuO NM inhalation exposure to asthmatic mice (comparison d): (a) percentages of cells in BAL fluid including eosinophils, neutrophils, lymphocytes, macrophages, and epithelial cells; (b) Percentages of each indicated subtype from parent cells (*see definition of parent cells in method 2.5*) in the lung tissue homogenates; (c) percentages of $CD44^{hi}$ $CD11a^{hi}$ $CD4^{+}$ T cells in lung tissue homogenates. Statistical analysis was performed using unequal variance unpaired T test (Welch T test). Data are shown as mean ±SE (n=6). ****P<0.0001, ***P<0.001, *P<0.05.

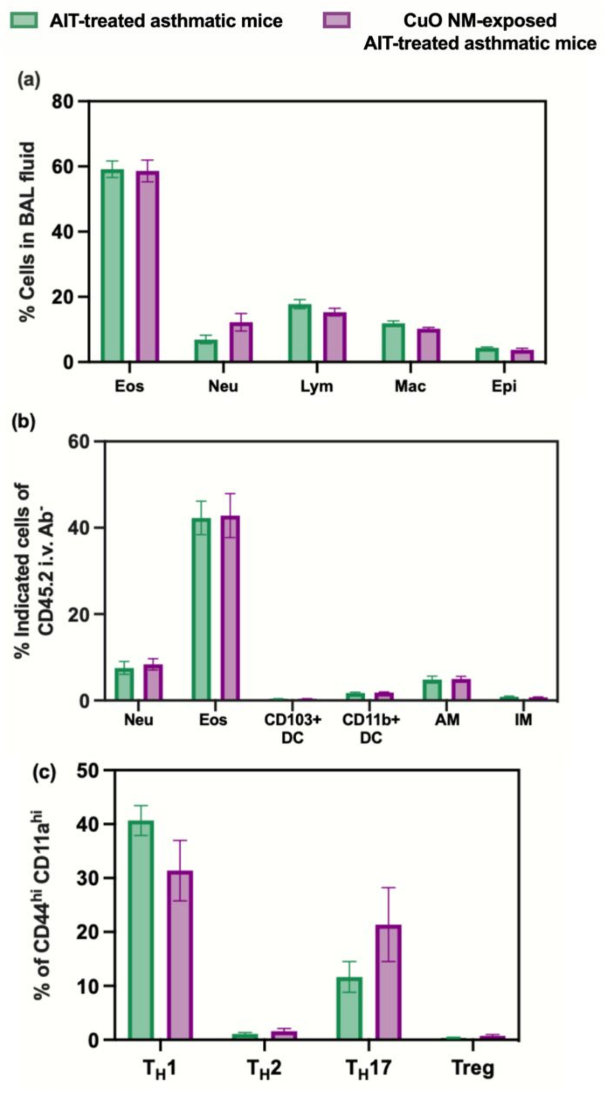

Figure S8. Assessment of immunomodulatory effects of CuO NM inhalation exposure to AIT-treated asthmatic mice (comparison e): (a) percentages of cells in BAL fluid including eosinophils, neutrophils, lymphocytes, macrophages, and epithelial cells; (b) Percentages of each indicated subtype from parent cells (*see definition of parent cells in method 2.5*) in the lung tissue homogenates; (c) percentages of $CD44^{hi}$ $CD11a^{hi}$ $CD4^{+}$ T cells in lung tissue homogenates. Statistical analysis was performed using unequal variance unpaired T test (Welch T test). Data were shown as mean ±SE (n=6). ****P<0.0001, ***P<0.001, *P<0.05.

Table S1. A list of surface staining antibodies and transcription factor staining antibodies for discrimination of antigen experienced $CD4^+$ T cell subsets including T_H1, T_H2, T_H17, and Treg cells

**Antibody Target**	**Fluorophore**	**Working concentration (µg/mL)**	**Clone**	**Company**
CD3	BV510	5	145-2C11	BioLegend
CD4	BV421	1	GK1.5	BioLegend
CD8a	BV786	1	53-6.7	BD Biosciences
CD11a	BB700	2	M17/4	BD Biosciences
CD49d	FITC	6.7	R1-2	BioLegend
CD44	AF700	2.5	IM7	BioLegend
CD25	PerCP	1	PC61	BioLegend
T-bet	PE-Cy7	1.25	eBio4B10	Thermofisher
GATA-3	BV711	5	L50-823	Thermofisher
Foxp3	PE	0.4	FJK-16S	Thermofisher
RORgt	BV650	2	Q31-378	BD Biosciences

Table S2. A list of surface staining antibodies for discrimination of neutrophils, eosinophils, macrophages, and dendritic cells.

Antibody Target	Fluorophore	Working concentration (μg/mL)	Clone	Company
CD86	BUV 805	2	24F	BD Biosciences
CD4	BV 421	1	GK1.5	BioLegend
Ly6G	BV 480	1	1A8	BD Biosciences
CD80	BV650	2	16-10A1	BD Biosciences
F4/80	BV 711	4	T45-2342	BD Biosciences
CD8	BV 786	1	53-6.7	BD Biosciences
Siglec F	BB 515	2	E50-2440	BD Biosciences
CD24	eFluor 450	2	M1/69	Thermofisher
CD11b	PE	0.4	HL3	BD Biosciences
CD64	PEDazzle 594	2	X54-5/7.1	BioLegend
CD11c	PE-Cy7	2	HL3	BD Biosciences
B220 (CD45)	PE-Cy5	2.5	RA3-6B2	BD Biosciences
MHC-II	AF 532	1	M5/114.15.2	Thermofisher
Ly6C	APC-fire 750	1	HK1.4	BioLegend
CD103	APC R700	2	M290	BD Biosciences
